# A first-of-its-kind two-body statistical shape model of the arthropathic shoulder: enhancing biomechanics and surgical planning

**DOI:** 10.1186/s13018-025-05855-4

**Published:** 2025-06-03

**Authors:** Justin Blackman, Joshua W. Giles

**Affiliations:** 1 Island Medical Program, Faculty of Medicine, University of British Columbia, University of Victoria, Victoria, BC Canada; 2 Department of Mechanical Engineering, University of Victoria, Victoria, BC Canada; 3 Department of Orthopaedics, University of British Columbia, Vancouver, BC Canada; 4 Institute On Ageing and Lifelong Health, University of Victoria, Victoria, BC Canada

**Keywords:** Statistical modeling, Shoulder arthroplasty, Surgical planning, Shape analysis, Computational orthopedics, Biomechanics, AI-CDSS

## Abstract

**Background:**

Statistical Shape Models are machine learning tools in computational orthopedics that enable the study of anatomical variability and the creation of synthetic models for pathogenetic analysis and surgical planning. Current models of the glenohumeral joint either describe individual bones or are limited to non-pathologic datasets, failing to capture coupled shape variation in arthropathic anatomy. We aimed to develop a novel combined scapula-proximal-humerus model applicable to clinical populations.

**Methods:**

Preoperative computed tomography scans from 45 Reverse Total Shoulder Arthroplasty patients were used to generate three-dimensional models of the scapula and proximal humerus. Correspondence point clouds were combined into a two-body shape model using Principal Component Analysis. Individual scapula-only and proximal-humerus-only shape models were also created for comparison. The models were validated using compactness, specificity, generalization ability, and leave-one-out cross-validation. The modes of variation for each model were also compared. The combined model was described using eigenvector decomposition into single body models. The models were further compared in their ability to predict the shape of one body when given the shape of its counterpart, and the generation of diverse realistic synthetic pairs de novo.

**Results:**

The scapula and proximal-humerus models performed comparably to previous studies with median average leave-one-out cross-validation errors of 1.08 mm (IQR: 0.359 mm), and 0.521 mm (IQR: 0.111 mm); the combined model was similar with median error of 1.13 mm (IQR: 0.239 mm). The combined model described coupled variations between the shapes equalling 43.2% of their individual variabilities, including the relationship between glenoid and humeral head erosions. The combined model outperformed the individual models generatively with reduced missing shape prediction bias (> 10%) and uniformly diverse shape plausibility (uniformity *p*-value < .001 vs. .59).

**Conclusions:**

This study developed the first two-body scapulohumeral shape model that captures coupled variations in arthropathic shoulder anatomy and the first proximal-humeral statistical model constructed using a clinical dataset. While single-body models are effective for descriptive tasks, combined models excel in generating joint-level anatomy. This model can be used to augment computational analyses of synthetic populations investigating shoulder biomechanics and surgical planning.

## Background

Statistical shape models (“SSMs”) [1] are machine learning applications to bony anatomy used to analyze the variability in anatomical geometry across a population. These models provide a compact representation of bone morphology and can be used in orthopaedics descriptively, for example to investigate the anatomical contributions to joint pathogenesis [2–4]. Alternatively, SSM can be used generatively, for example to produce large sets of realistic synthetic shapes for computational analysis of surgical intervention for joint reconstruction [5, 6]**.** Previous research has demonstrated the necessity of shoulder arthroplasty technique adjustment in light of variation in glenohumeral morphology [7, 8]; SSMs empower the underlying biomechanical studies that inform the optimal adjustment of prosthesis selection and placement for an individual patient. SSMs leverage artificial intelligence to identify complex patterns in bone morphology across populations, enabling patient categorization in novel and potentially more powerful ways than traditional methods, which are inherently constrained by human-driven, low-dimensional assessments.

Single-body SSMs for individual anatomical structures, such as the scapula [9–11] and the humerus [12, 13], among others, are common in the literature. These models effectively describe modes of variation, achieving good generalizability and strong correspondence with clinically relevant anatomical measurements. However single-body SSMs, even if using two models to describe both the scapula and humerus [14, 15], are inherently limited in their scope as these models focus on local morphology and fail to analyze anatomical relationships (dependence) at the joint level [16]. Independent Scapula and Humerus SSMs that were developed in parallel have demonstrated significant correlation between the two bodies [14], but do not quantify how these correlations couple along unified modes of variation, for which a combined two-body SSM is necessary.

Multi-body SSMs have been developed to analyze relationships between multiple anatomical structures [17, 18], including the scapula and humerus, some with an emphasis on relative positioning between bones [19, 20]. However, these models were predominantly trained on non-pathologic scans and are thus limited in their applicability to clinical populations undergoing arthroplasty procedures. SSMs of arthropathic specimens are exceedingly rare, with one previous scapula model constructed using clinical patient datasets [9], and no such humeral models to date. No two-body SSM that captures the simultaneous coupled variation of the scapula and proximal humerus in populations requiring Reverse Total Shoulder Arthroplasty (“RTSA”) has been published to date.

The primary objectives of this study were twofold. First, to develop and validate a combined SSM of the scapula and proximal humerus that captures coupled variation of the two bodies and is useful in surgical planning, and describing pathologic shoulder anatomy. Second, to develop an analysis framework of the combined two-body scapula-proximal-humerus SSM that intuitively characterizes its utility in descriptive and generative capacities compared to independent one-body SSMs. This work provides a primer on statistical shape modeling and the extension into multi-domain shape models.

## Methods

All numerical methods and visualizations were implemented in Python 3.9.13 [21–25].

### Subject 3D volume representations

Preoperative CT scans were obtained from a heterogeneous patient cohort ($n=45$) who subsequently underwent RTSA. Subject scans were reviewed to ensure the inclusion of the distal deltoid insertion at the deltoid tuberosity of the humerus. This landmark was used to define the distal termination point for the proximal humerus, ensuring anatomical uniformity across study subjects. Demographic data for the study population is provided in Table 1.

**Table 1:** Demographic data of all subjects in the training set

Sex/Side	Quantity	Age (Mean ± SD)
Male	22	71.8 ± 5.69
Female	23	72.2 ± 6.75
Left	18	70.3 ± 7.56
Right	27	73.1 ± 4.67
All	45	71.9 ± 6.27

The proximal humerus, rather than the entire humerus, was selected for this study based on its clinical relevance given that the full humerus is not included in a conventional pre-operative arthroplasty imaging series of the shoulder [26, 27]. Thus, clinical populations typically have preoperative computed tomography (“CT”) scans [28] that capture the proximal humerus, but not the distal portion. As a result, focusing on the proximal humerus aligns with real-world clinical workflows and ensures the model reflects the data most commonly encountered in practice. Furthermore, the shape of the entire humerus can often be inferred from the proximal humeral morphology, as it carries much of the biomechanical and anatomical information needed for joint-level analysis [29, 30]. Including the deltoid insertion as a distal landmark ensures the incorporation of critical biomechanical details relevant to shoulder function and surgical planning.

Three-dimensional volumetric models of the scapulae and proximal humeri were generated for all subjects through manual segmentation using the commercial software, Mimics version 23 (Materialise, Leuven, Belgium). The segmentation outputs were remeshed to achieve uniform 1 mm edge-length triangular surface meshes using commercial software, 3-matic version 15 (Materialise, Leuven, Belgium). Additionally, to standardize the data, left-sided scapulae and proximal humeri were reflected across the sagittal plane, resulting in a dataset consisting exclusively of right-sided appearing bony anatomy. Radiographic morphological characteristics of interest for the study cohort are provided in Table 2.

**Table 2:** Morphologic data across subjects in the training set

Dimension	Mean	SD
Scapula Height (mm) [9]	154	11.8
Scapula Width (mm) [9]	104	7.63
Scapula Aspect Ratio (Width/Height)	0.673	0.0410
Glenoid Height (mm) [9]	39.5	4.11
Glenoid Width (mm) [9]	29.1	3.48
Acromion Length (mm) [9]	45.7	6.45
Lateral Acromion to Glenoid Center (mm) [9]	28.4	5.05
Coracoid Tip to Glenoid Center (mm) [9]	16.3	4.19
Posterior-Inferior Acromion to Glenoid Center (mm) [9]	40.1	4.55
Superior-Anterior Acromion to Glenoid Center (mm) [9]	6.26	5.46
Fulcrum Axis (°) [9]	94.1	3.12
Glenoid Inclination Angle (°) [9]	97.6	4.15
Glenoid Version Angle (°) [9]	94.1	7.57
Acromial Tilt Angle (°) [9]	32.4	3.58
Critical Shoulder Angle (°) [9]	29.4	5.02
Superior-Inferior Glenoid-Acromion Angle (°) [9]	52.4	7.70
Superior-Inferior Glenoid-Acromion-Coracoid Angle (°) [9]	95.4	4.57
Proximal Humeral Length (mm)	154	10.3
Humeral Shaft Diameter (mm) [12]	22.4	2.50
Humeral Head Radius of Rotation (mm) [31]	23.3	2.20
Humeral Head Inclination Angle (°) [32]	134	7.60
Humeral Head Medial Offset (mm) [33]	0.261	3.83
Humerus Greater Tuberosity Angle (°) [34]	64.1	5.00
Posterior Static Subluxation of the Humerus (mm) [31]	6.32	2.56

### Centred and aligned point correspondences

ShapeWorks Studio [35] software was used to develop point correspondences across the subject scapulae and proximal humeri; the set of scapulae and set of proximal humeri were processed separately to allow for different optimization parameters based on the distinct needs of each bone set (Table 3).

**Table 3:** Parameters used for the ShapeWorks Studio point correspondence optimizations

Optimization Parameter	Scapulae	Humeri
Grooming Alignment	Landmarks	Landmarks
Number of Landmarks	25	3
Number of Particles	1024	1024
Initial Relative Weighting	0.3	0.05
Relative Weighting	6	1
Starting Regularization	500	1000
Ending Regularization	1	10
Iterations per Split	500	1000
Optimization Iterations	500	1000
Geodesic Distance?	Yes	No
Geodesic Remesh	100%	N/A
Normals?	Yes	No
Normals Strength	7	N/A
Use Initial Landmarks?	Yes	Yes
Narrow Band	4	4

**Fig. 1:**
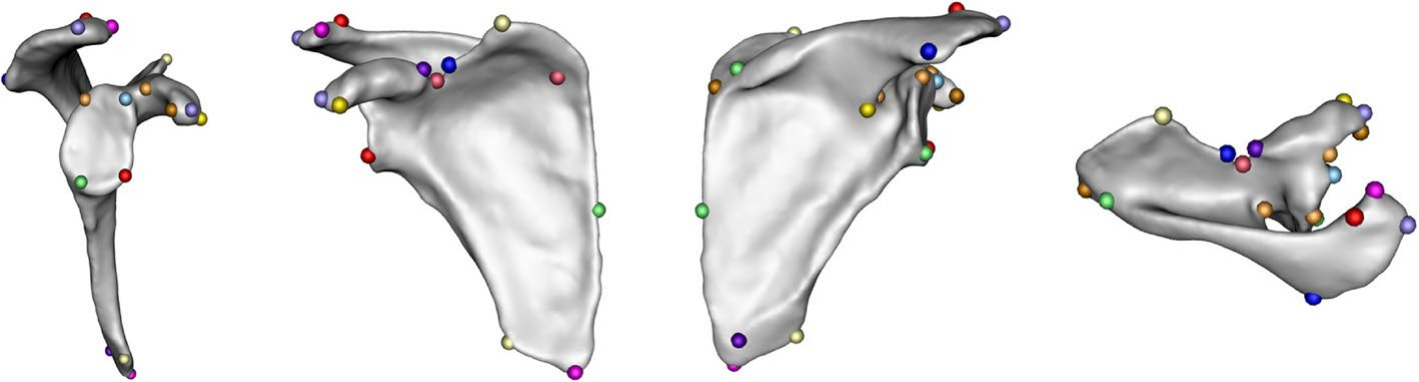
Scapula landmarks — lateral, anterior, posterior, and superior views

**Fig. 2:**
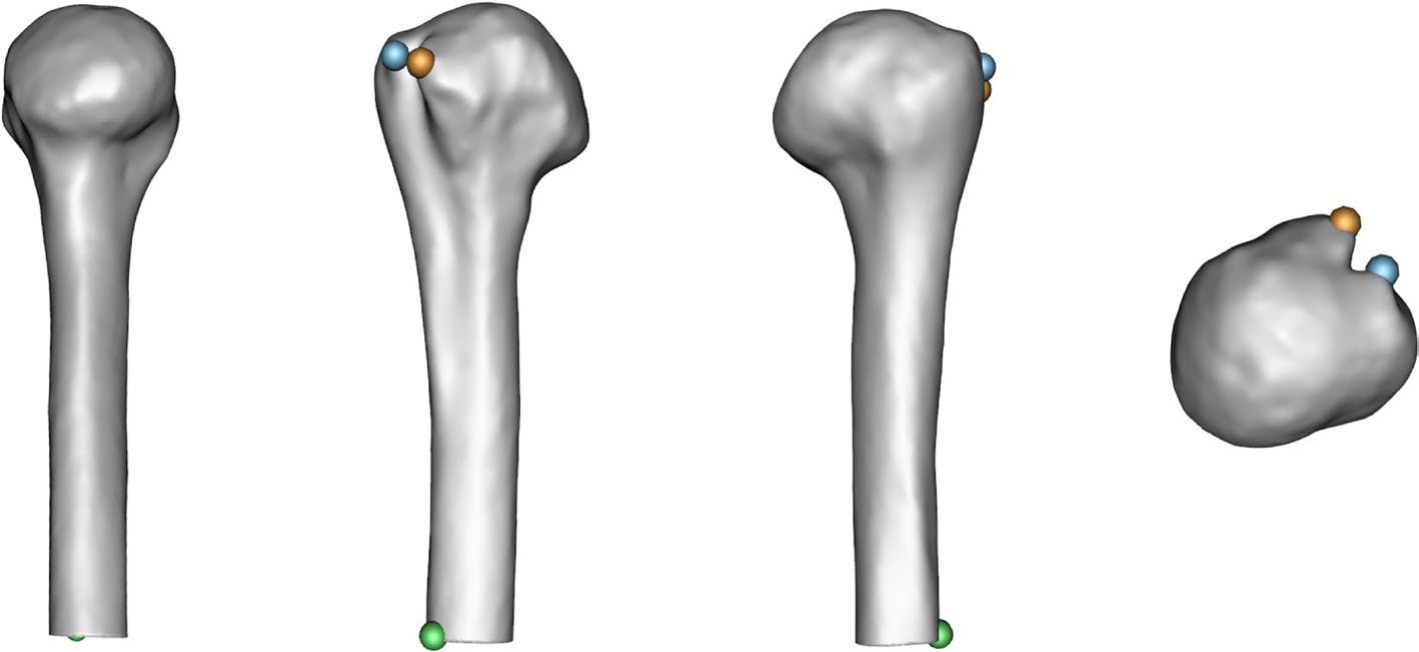
Humerus landmarks — medial, anterior, posterior, and superior views

The 3D volume representations were first groomed in ShapeWorks Studio [36] by centering to a common origin, and then aligned using manually placed landmarks (Figs. 1 and 2, Tables 4 and 5).

**Table 4:** Scapula landmark descriptions

Landmarks		
Antero-Superior Glenoid Rim	Anterior Acromion	Inferior Infraspinatus Fossa
Postero-Superior Glenoid Rim	Lateral Acromion	Postero-Inferior Base of Spine
Antero-Inferior Glenoid Rim	Medial Acromion	Medio-Superior Subscapular Fossa
Postero-Inferior Glenoid Rim	Posterior Acromion	Medial Trapezius Surface of Spine
Anterior Coracoid	Superior Angle	Trigonum Spinae
Lateral Coracoid	Inferior Angle	Mid-Medial Border
Posterior Coracoid	Nadir of Suprascapular Notch	Lateral Projection of Axillary Border
Medial Suprascapular Notch	Lateral Superior Scapular Notch	
Apex of Coracoid Concavity	Spinoglenoid Notch

**Table 5:** Humerus landmark descriptions

Landmarks
Antero-Lateral Lesser Tuberosity
Antero-Medial Greater Tuberosity
Distal Greater Tuberosity Ridge

The same landmarks were also used for correspondence point initialization. The difference in the number of landmarks used for the scapulae compared to the proximal humeri is due to the relative complexity of the scapular shape, which required substantially more landmarks to achieve acceptable correspondence point dispersion across the shape. Correspondence point cloud placement optimizations resulted in $n=45$ corresponding scapula point-clouds, each with 1600 points defined by three-dimensional coordinate sets $\{({x}_{i } , {y}_{i} , {z}_{i})| i\,\epsilon\,{\mathbb{N}}\le 1600\}$ and $n=45$ corresponding proximal-humerus point clouds, each with 1536 points defined by three-dimensional coordinate sets $\{({x}_{i }, {y}_{i} , {z}_{i})| i\,\epsilon\,{\mathbb{N}}\le 1536\}$.

### Matrix representation of correspondence point clouds

The scapula point clouds were represented as vectors through simple concatenation of the point coordinates, $S = \{ {s}_{j }= [{x}_{1},{y}_{1},{z}_{1},{x}_{2},{y}_{2},{z}_{2}, ... , {x}_{m}, {y}_{m},{z}_{m}{]}^{T}|m =1600, j\,\epsilon\,{\mathbb{N}}\le 45 \}$. Similarly, the proximal-humerus point clouds were represented as the set of vectors $H = \{ {h}_{j }= [{x}_{1},{y}_{1},{z}_{1},{x}_{2},{y}_{2},{z}_{2}, ... , {x}_{m}, {y}_{m},{z}_{m}{]}^{T}|m =1536, j\,\epsilon\,{\mathbb{N}}\le 45 \}$. Matching scapula and proximal-humerus pairs were also combined through simple concatenation to produce a set of two-body corresponding point-cloud vectors, $C = \{ {c}_{j }= [{s}_{j}, {h}_{j}{]}^{T}|j\,\epsilon\,{\mathbb{N}}\le 45 \}$, with each ${c}_{j}$ a $3m=3(1600+1536) =9408$ element vector. Each set $S$, $H$, and $C$ was then represented as a matrix through columnar concatenation of the set’s constituent vector elements, $X \to X = [ {x}_{1}, {x}_{2}, ... , {x}_{n} ]$.

### (ℓ2-norm) principal component analysis

Principal Component Analysis (“PCA”) [37] proceeded on each of $S$, $H$, and ${\varvec{C}}$ (or variants thereof when withholding subjects from the training sets in accordance with the following sections) in the usual fashion with the computation of the set’s covariance matrix, and the subsequent eigenvector decomposition thereof. The data on which the PCA was performed was not preprocessed by scaling, to maintain overall size variation information in the model. The data was centred such that each scapula and each proximal humerus has its centroid at the origin; centering consists of a three dimensional translation that does not affect shape and ensures that PCA captures shape and not position (or “pose” [19]) variation. A training set of $n$ samples will yield only $n-1$ non-trivial eigenvectors, which can be explained by understanding the training set mean as the $n$-th degree of freedom in the system. Analogously, the span of two points in three-dimensional space can be defined by a (one-dimensional) vector and an offset, the span of three points in three-dimensional space can be defined by a (two-dimensional) plane and an offset, and so on. PCA results in sorted matrices of eigenvectors, ${\Phi }_{S}$, ${\Phi }_{H}$ and ${\Phi }_{C}$, where each column in a matrix forms an eigenvector (or “principal component” or “mode of variation”) with corresponding eigenvalue greater than the corresponding eigenvalues of all eigenvectors to the right of it, ${\Phi }_{X} = [{\varphi }_{1} ,{\varphi }_{2} , ... , {\varphi }_{n-1} ]$, where ${\varphi }_{i}\leftrightarrow {\lambda }_{i} , {\lambda }_{i}\ge {\lambda }_{i+1}$.

The matrices of sorted eigenvectors ${\Phi }_{S}$, ${\Phi }_{H}$ and ${\Phi }_{C}$ paired with their accompanying training set means $\overline{{{\varvec{x}} }_{{\varvec{S}}}}$, $\overline{{{\varvec{x}} }_{{\varvec{H}}}}$ and $\overline{{{\varvec{x}} }_{{\varvec{C}}}}$ comprise three distinct SSMs, describing shape variations in scapulae, proximal humeri, and both scapulae and proximal humeri simultaneously, respectively. That is to say, given an eigenvector matrix $\Phi$, its corresponding eigenvalues ${\lambda }_{i}$, and the training data matrix $X$, we can produce new shapes that are similar to the original set using $y =\overline{x }$ + $\Phi {\varvec{b}}$, where $\overline{x } =\left\{[ \overline{{x }_{1}},\overline{{x }_{2}}, ... ,\overline{{x }_{3m}}{]}^{T} |\overline{{x }_{i}}=\frac{1}{n}{\sum }_{j=1}^{n}{X}_{i,j}\right\}$, and where we can assume ${b}_{i} \sim N(0,{\lambda }_{i})$ such that ${\sum }_{i=1}^{n-1}\frac{{{b}_{i}}^{2}}{{\lambda }_{i}}\le {\chi }_{\alpha , n-1}^{2}$ for desired certainty level α [38].

### Distance measures

A suitable metric with which to measure the difference between two point clouds is necessary in order to assess the performance of the generated SSMs. For example, there is a need to quantify the *error* of a generated model output, itself a point cloud or equivalently a vector, ${o}= [{x}_{o,1},{y}_{o,1},{z}_{o,1},{x}_{o,2},{y}_{o,2},{z}_{o,2}, ... , {x}_{o,m}, {y}_{o,m},{z}_{o,m}{]}^{T}$, and the target point cloud attempted at being represented by the SSM, ${t}= [{x}_{t,1},{y}_{t,1},{z}_{t,1},{x}_{t,2},{y}_{t,2},{z}_{t,2}, ... , {x}_{t,m}, {y}_{t,m},{z}_{t,m}{]}^{T}$ (i.e. error) denoted as $d(o,t)$.

The Root Mean Square (RMS) difference between vectors is often selected as the distance measure in the evaluation of SSMs [9, 11, 14, 18, 29], both for consistency with the interpretation of ℓ2-norm PCA as a minimization of a sum of squared errors of vector elements, and the relative ease of calculation:

$$RMS({\varvec{o}},{\varvec{t}})=\sqrt{\frac{1}{3m}{\sum }_{i=1}^{3m}({o}_{i}-{t}_{i}{)}^{2}}=\frac{1}{\sqrt{3m}}\sqrt{{\sum }_{i=1}^{m}\left(({x}_{o,i}-{x}_{t,i}{)}^{2}+({y}_{o,i}-{y}_{t,i}{)}^{2}+({z}_{o,i}-{z}_{t,i}{)}^{2}\right)}.$$

However, this metric does not have a direct intuitive interpretation as to how the value translates to errors in model representations in 3D space. For this reason, the average Euclidean point distance between point clouds was instead selected as a distance measure:

$$d({\varvec{o}},{\varvec{t}})= \frac{1}{m}{\sum }_{i=1}^{m}\sqrt{({x}_{o,i}-{x}_{t,i}{)}^{2}+({y}_{o,i}-{y}_{t,i}{)}^{2}+({z}_{o,i}-{z}_{t,i}{)}^{2}}.$$

Care must be taken when comparing results reporting $d(o,t)$ with those reporting $RMS(o,t)$, as typically $d(o,t) >RMS(o,t)$ given Jensen’s inequality [39] for concave $f(x) = \sqrt{x}$.

The Hausdorff metric [40, 41], the longest Euclidean distance between nearest neighbours across two point clouds, was selected as a measure of worst-case distance as is convention in the literature of computer vision and SSMs [9, 11, 15, 42, 43]. Considering each point cloud vector a set of $m$ points, and each point itself a vector of 3D coordinates, $s=\{ s=[{x}_{i }, {y}_{i}, {z}_{i}]| i\,\epsilon\,{\mathbb{N}}\le m\}$, the following (bidirectional) Hausdorff metric is used [44]:

$${d}_{H}({\varvec{o}},{\varvec{t}})=max\left[ma{x}_{o \,\epsilon\, {\varvec{o}}}\left\{mi{n}_{t \,\epsilon\, {\varvec{t}} } d(o,t)\right\} , {max}_{t \,\epsilon\, {\varvec{t}}}\left\{mi{n}_{o \,\epsilon\, {\varvec{o}} } d(o,t)\right\}\right]$$

### Model descriptive ground truth accuracy

The *Ground Truth Accuracy* [45] of the generated SSMs was quantified via leave-one-out cross-validation. This test assesses the ability of the SSMs to describe a single shape outside of their training sets, and was repeated to iteratively exclude and subsequently model each of the $n=45$ subjects from the data sets. Thus, the results for each SSM form a distribution of $n=45$ test observations.

For each test, each of the three SSMs is regenerated using a modified training set, ${X}_{i}$, that excludes the $i$-th subject${x}_{i}$. We generate a parameter vector of best fit using all $\left(n-1\right)-1=43$ resulting non-trivial eigenvectors as principal components, $b ={{\Phi }^{+}(x}_{i}-\overline{{x }_{i}})$, where $\overline{{x }_{i}}$ is the average shape of the training set ${X}_{i}$. Here ${\Phi }^{+}$ is the transpose of the eigenvector matrix (as $\Phi {\Phi }^{T}={I}_{n}$ by virtue of its semi-unitary semi-orthogonality and dimensions resulting in left-invertibility [46]). To ensure plausibility of the SSM representation in alignment with the assumption of ${b}_{i} \sim N(0,{\lambda }_{i})$, the resulting $b$ values are regulated, first by scaling individual elements such that $\left|{b}_{i}\right|\le 3\sqrt{{\lambda }_{i}}$, and subsequently by applying the uniform scaling factor $s$ closest to $1$ such that ${\sum }_{i=1}^{n-1}\frac{{({s \times b}_{i})}^{2}}{{\lambda }_{i}}\le {\chi }_{0.997, n-1}^{2}$, resulting in $\widehat{b}$ that resides within the Mahalanobis hyperellipsoid [47] defining the region of acceptable plausibility [38]. The determination of $s$ was implemented as a constrained quadratic programming [48] optimization problem minimizing $(s-1{)}^{2}$. The SSM representation ${y}_{i}$ of the target shape ${x}_{i}$, is then calculated ${y}_{i} = \overline{{x }_{i}}$ + $\Phi \widehat{{\varvec{b}}}$ for which the average Euclidean point distance $d({y}_{i},{x}_{i})$ and Hausdorff metric ${d}_{H}({y}_{i},{x}_{i})$ are measured. The Euclidean point distance and Hausdorff metric between the training set average $\overline{{x }_{i}}$ and the target left-out sample ${x}_{i}$ are also calculated to compare the extent of SSM prediction accuracy that is attributable to the inclusion of the simple mean versus the inclusion of the weighted principal components.

### Model generalisation ability

The concept of model *Ground Truth Accuracy* is generalised by assessing the ability of an SSM to describe a single shape outside of its training set while simultaneously restricting either the number of samples included in the training set or the number of principal components included in the SSM. At the extreme, when including a full training set of $n-1$ samples and all available $n-2$ non-trivial principal components (given that one training set sample is left out), the measures of *Generalisation Ability* reduce to the model *Ground Truth Accuracy*; however by restricting these model design criteria, we gain insight into the sufficiency of model training set sizes and the model tolerances for dimensional reduction.

#### Model generalisation ability: number of training samples

For each SSM, *Generalisation Ability* for number of training samples [45] is assessed through leave-one-out cross-validation calculating the average Euclidean point distance between the left-out target and the model-generated shape, repeated across training sample sizes $k=\{\text{2,3}, ..., n-1\}$, and iterated over the entire data set for each training sample size; each cross validation instance trains the SSMs on $k$ randomly selected samples using all $k-1$ non-trivial principal components. Thus, the result for each SSM at each training set size is a distribution of $n=45$ test observations.

### Model generalisation ability: number of principal components

For each SSM, *Generalisation Ability* for number of principal components [45] is assessed through leave-one-out cross-validation calculating the average Euclidean point distance between the left-out target and the model-generated shape, repeated across number of eigenvectors included as principal components $k=\{\text{1,2},3, ..., n-2\}$, and iterated over the entire data set for each number of principal components. Thus, the result for each SSM at each number of principal components is a distribution of $n=45$ test observations.

### Model specificity

The propensity of an SSM to produce only *realistic* shapes that are similar to the original training set is termed model *Specificity* [45]. Each SSM is trained on the entire data set of $n=45$ samples, then a large set of $M=\text{10,000}$ generated shapes are created by generating parameter vectors $b$ with randomly sampled ${b}_{i} \sim N(0,{\lambda }_{i})$ and iterating the entire process across a varying number of principal components included in the SSM, $k=\{\text{1,2},3, ..., n-1\}$. The results for each SSM at each number of principal components are distributions of $M$ observations of the average Euclidean point distances and Hausdorff metrics between the generated shapes and the closest shapes within the training set.

### Model compactness

SSM *Compactness* explores the number of eigenvectors required as principal components included in an SSM in order to describe a desired amount of variation observed within the training set [45]. Each constituent principal component has a corresponding eigenvalue ${\lambda }_{i}$, itself a quantification of the observed training set variation explained by the principal component. For a given number of principal components, $k$, we define Compactness of an SSM described by eigenvector matrix ${\Phi }_{X}$ as the proportion of total training set variance described by the first $k$ principal components: $c(\Phi ,k)=\frac{{\sum }_{i=1}^{k}{\lambda }_{i}}{{\sum }_{i=1}^{n-1}{\lambda }_{i}}$. Compactness plots are produced by varying $k\in {\mathbb{N}}\le n-1$.

### SSM modes of variation

The range of shapes occurring along one of the $n-1$ modes of variation of an SSM, ${\varphi }_{i}$, can be investigated by starting with the average shape across the training set $\overline{{\varvec{x}} }$, and generating model outputs ${{\varvec{y}}}_{i,j} = \overline{{\varvec{x}} }$ + ${\boldsymbol{\varphi }}_{i}{b}_{i,j}$ while sweeping across the feasible range of the corresponding model parameter, $-3\sqrt{{\lambda }_{i}}\le {b}_{i,j}\le 3\sqrt{{\lambda }_{i}}$.

This analysis provides insight into the shape variations that are characteristic of the modelled bone across the study population, the relative importance of each shape variation in describing the heterogeneity of the study population, and a point of comparison between this work and previous scapular and proximal-humeral SSMs.

### Quantification of coupled scapulohumeral variation

An understanding of the characteristic variations described by the single-body SSMs can be applied to the Combined scapula and proximal-humerus SSM by expressing the principal components of the Combined SSM as linear combinations of the principal components of the single-body SSMs. This decomposition also facilitates addressing the utility of a combined two-body model as opposed to using two independent single-body models when generating synthetic scapulohumeral shapes.

Given that the scapula and humerus data set vectors are of the form $[{x}_{1},{y}_{1},{z}_{1},{x}_{2},{y}_{2},{z}_{2}, ... , {x}_{m}, {y}_{m},{z}_{m}{]}^{T}$ and the combined data set vectors are in turn of the form$[{s}_{j }, {h}_{j}{]}^{T}$, it follows that eigenvectors of the combined SSM are of the form ${\left[{\phi }_{S},{\phi }_{H}\right]}^{T}$ such that the combined SSM eigenvector matrix ${\Phi }_{C}$ consists of an upper portion ${\Phi }_{C,S}$ relating to the scapula and a lower portion ${\Phi }_{C,H}$ relating to the humerus, which can be decomposed separately.

For each constituent eigenvector in ${\boldsymbol{\Phi }}_{C,S}$ (denoted ${\boldsymbol{\varphi }}_{C,S,j}$) we seek a linear combination of constituent eigenvectors in ${\boldsymbol{\Phi }}_{S}$ defined as vector ${{\varvec{\gamma}}}_{j}$, such that ${\boldsymbol{\varphi }}_{C,S,j}={{\boldsymbol{\Phi }}_{S}}^{T}{{\varvec{\gamma}}}_{j} \Rightarrow {{\varvec{\gamma}}}_{j} ={{\boldsymbol{\Phi }}_{S}}^{+}{\boldsymbol{\varphi }}_{C,S,j}$, where ${{\boldsymbol{\Phi }}_{S}}^{+}$ is simply the eigenvector matrix (as $\boldsymbol{\Phi }{\boldsymbol{\Phi }}^{{\varvec{T}}}={{\varvec{I}}}_{n}$ by virtue of its semi-unitary semi-orthogonality and dimensions resulting in left-invertibility).

The single-body eigenvector linear combination weights ${\gamma }_{j}$ are converted into directionless (absolute) proportions of contribution magnitude to the resulting combined model eigenvector ${\boldsymbol{\varphi }}_{C,S,j}$ by ${\boldsymbol{\alpha }}_{S,j}=\left[{\alpha }_{S,i}=\frac{\left|{\gamma }_{{j}_{i}}\right|}{{\sum }_{k=1}^{n-1}\left|{\gamma }_{{j}_{k}}\right|}\right]$ where ${{\gamma }_{j}}_{i}$ denotes the $i$-th element of the vector ${{\varvec{\gamma}}}_{j}$. The resulting ${{\boldsymbol{\alpha }}_{S,j}}^{T}$ vectors are row-concatenated to form weighting matrix ${{\varvec{A}}}_{S}$ which can subsequently be visualised through plotting as a heatmap; the $i,j$-th element of ${{\varvec{A}}}_{S}$ corresponds to the proportion of contribution of the $j$-th scapula single-body SSM eigenvector to the scapular component of the $i$-th Combined SSM eigenvector.

Noting that the eigenvalues resulting from PCA encode the proportion of total training set variance explained by the accompanying eigenvector by $\frac{{\lambda }_{i}}{{\sum }_{j=1}^{n-1}{\lambda }_{j}}$ [37], and that the variance of a linear combination of *orthogonal* elements $Y = {\alpha }_{1}{X}_{1}+ ... +{\alpha }_{m}{X}_{m}$ is ${{\alpha }_{1}}^{2}VAR[{X}_{1}]+ ... + {{\alpha }_{m}}^{2}VAR[{X}_{m}]$ [49], we can calculate the proportion of scapula dataset variance described by the $j$-th principal component of the Combined SSM as ${v}_{C,S,j}={{{\varvec{\gamma}}}_{j}}^{*2}{{\varvec{\lambda}}}_{s}$ where ${{{\varvec{\gamma}}}_{j}}^{*2}$ denotes item-wise squared vector, and ${{\varvec{\lambda}}}_{s}$ is a vector of the proportions of total scapula training set variance described by each Scapula SSM principal component.

The constituent eigenvectors of ${\boldsymbol{\Phi }}_{C,H}$ are decomposed into linear combinations of the constituent eigenvectors of ${\boldsymbol{\Phi }}_{H}$, processed into ${{\varvec{A}}}_{H}$, and used to calculate ${v}_{C,H,j}$, analogously.

The coupled variations for single body shapes that coincide in one principal component of the Combined SSM, ${\boldsymbol{\varphi }}_{C,i}$, are then given by ${v}_{J,j}= min\left({v}_{C,S,j} , {v}_{C,H,j}\right)$.

### Prediction of missing counterpart

The consideration of a two-body Combined SSM rather than two independent single-body SSMs naturally leads to the question of model prediction accuracy in contexts where only one body is provided (e.g. in medical imaging) and the shape of the matching second body must be predicted. With the two independent single-body SSMs, no coupled variation between the two bodies is modeled, and so the missing body can only be estimated as the training set mean, $E({{\varvec{x}}}_{2,i}|{{\varvec{x}}}_{1,i}) =\overline{{{\varvec{x}} }_{2}}$ [50].

However, the two-body Combined SSM can be assessed for coupled variation between the two bodies; if the coupled variation is present, it may be used to improve the prediction accuracy beyond that of the training set mean. Specifically, recalling that Combined SSM model parameters are calculated as ${{\varvec{b}}}_{i} ={\boldsymbol{\Phi }}_{C}^{T}({{\varvec{x}}}_{i}-\overline{{\varvec{x}} })$ and noting that ${\boldsymbol{\Phi }}_{C}$, ${{\varvec{x}}}_{i}$ and $\overline{{\varvec{x}} }$ can each be decomposed into constituent scapular and proximal humeral components, results in the following two-body decomposition for Combined SSM Model parameters:

$${{\varvec{b}}}_{{\varvec{i}}}={{\varvec{b}}}_{S,i} +{{\varvec{b}}}_{H,i} =={\boldsymbol{\Phi }}_{C,S}^{T}({{\varvec{x}}}_{S,i}-\overline{{{\varvec{x}} }_{S}})+{\boldsymbol{\Phi }}_{C,H}^{T}({{\varvec{x}}}_{H,i}-\overline{{{\varvec{x}} }_{H}}).$$

Linear covariation between ${b}_{S}$ and ${b}_{H}$ can be assessed through the covariance matrix (the derivation of which is omitted for brevity), $Cov({{\varvec{b}}}_{S},{{\varvec{b}}}_{H})=\left({\boldsymbol{\Phi }}_{C,S}^{T}{ \boldsymbol{\Sigma }}_{S,H}{\boldsymbol{\Phi }}_{C,H}\right)$, where $$Cov({{\varvec{x}}}_{S},{{\varvec{x}}}_{H})=\left[\begin{array}{cc}{\boldsymbol{\Sigma }}_{S,S}& {\boldsymbol{\Sigma }}_{S,H}\\ {\boldsymbol{\Sigma }}_{H,S}& {\boldsymbol{\Sigma }}_{H,H}\end{array}\right]$$ [51]. The relative magnitude of covariance between the $i$-th scapular model component contribution and the $j$-th proximal-humeral model component contribution to the Combined SSM model parameters across the training set, ${{\varvec{A}}}_{B}$, can be visualised by a heatmap of the absolute values of the covariance, standardised to the maximum absolute element in the covariance matrix.

In light of the above decomposition, the naive estimate for the missing shape (i.e. the training set mean shape), $E({{\varvec{x}}}_{2,i}|{{\varvec{x}}}_{1,i}) =E({{\varvec{x}}}_{2,i}) = \overline{{{\varvec{x}} }_{2}}$ can be understood as assuming ${{\varvec{b}}}_{2,i}\approx 0$, since ${{\varvec{x}}}_{2,i} ={\boldsymbol{\Phi }}_{C,2}\left({{\varvec{b}}}_{i} -{{\boldsymbol{\Phi }}_{C,1}}^{T}({{\varvec{x}}}_{1,i}-\overline{{{\varvec{x}} }_{1}})\right)+\overline{{{\varvec{x}} }_{2}}$ and therefore ${{\varvec{b}}}_{i}-{{\varvec{b}}}_{1,i}=0$. With a Combined SSM, in the event of correlation between ${{\varvec{b}}}_{1,i}$ and ${{\varvec{b}}}_{2,i}$, ${{\varvec{b}}}_{2,i}$ can instead be predicted from ${{\varvec{b}}}_{1,i}$, for example through a form of regression, as $E({{\varvec{x}}}_{2,i}|{{\varvec{x}}}_{1,i}) =\overline{{{\varvec{x}} }_{2}}$ + ${\boldsymbol{\Phi }}_{C,2}E({{\varvec{b}}}_{2,i}|{{\varvec{b}}}_{1,i})$.

Leave-one-out cross-validation is used to quantify the relative accuracy in predicting the shape of missing bodies between the independent single-body SSMs and the Combined SSM. The SSMs are first trained on $n-1$ paired scapula-proximal-humerus samples, and then predict the shape of a missing scapula based on the corresponding proximal humerus, and then vice versa. The independent single-body SSMs predict the missing bodies using the training set means, and the Combined SSM predicts the missing body by calculating ${{{\varvec{b}}}_{1,i}= \boldsymbol{\Phi }}_{C,1}^{T}({{\varvec{x}}}_{1,i}-\overline{{{\varvec{x}} }_{1}})$, using a Random Forest Regressor to predict ${{\varvec{b}}}_{2,i}$ from ${{\varvec{b}}}_{1,i}$, filtering the predicted ${{\varvec{b}}}_{2,i}$ into $\widehat{{{\varvec{b}}}_{2,i}}$ using the same procedure as for the *Ground Truth* and *Generalisation* tests, and then generating a shape prediction ${{\varvec{y}}}_{2,i} = \overline{{{\varvec{x}} }_{2,i}}+ {\boldsymbol{\Phi }}_{C,2} \widehat{{{\varvec{b}}}_{2,i}}$. The results for the two methods form distributions of $n=45$ average Euclidean point distance and Hausdorff metric observations for the scapula and proximal humerus.

### De Novo generation of synthetic populations

Synthetic populations for computational analyses must consist of individuals that are sufficiently realistic, while collectively capturing the entire range of possible variations expected within the emulated population. In other words, an unbiased generated population would produce a uniform distribution when evaluated through the population’s cumulative distribution function (if known). This is the property that is exploited in Inverse Transform Sampling [52], which is effectively the opposite process of how we proceed. We define the *plausibility* of a shape as the probability of observing a shape that is *more* extreme than it, calculated as 1 minus the cumulative distribution function value for the shape.

When generating synthetic shapes from an SSM the model parameters are often constrained, such as $\left|{b}_{i}\right|\le 3\sqrt{{\lambda }_{i}}$, due to the underlying assumption of independent ${b}_{i} \sim N(0,{\lambda }_{i})$ [45]. As a result of this assumption, $\sum \frac{{{b}_{i}}^{2}}{{\lambda }_{i}}\le {\chi }_{\alpha , n-1}^{2}$ [47] and therefore the probability of obtaining a set of model parameters $b$ less than a particular $\widehat{b}$ is given by the cumulative distribution function ${F}_{{\chi }^{2}}\left(\sum \frac{{{\widehat{b}}_{i}}^{2}}{{\lambda }_{i}},n-1\right)$ [38]. To assess for relative bias in the generation of synthetic shapes when using two independent single-body SSMs compared to using a single Combined SSM, a large set of scapula and proximal humerus shapes is generated by sampling from ${b}_{S,i} \sim N(0,{\lambda }_{S,i})$ and ${b}_{H,i} \sim N\left(0,{\lambda }_{H,i}\right)$ $M=\text{10,000}$ times, fitting Combined SSM model parameters ${b}_{C,i}$ to each generated shape pair, and computing the *plausibility* ($1-{F}_{{\chi }^{2}}$) for each shape pair. The plausibility is an inverse measure of a generated shape’s deviation from the training set mean. Similarly, $M=\text{10,000}$ ${b}_{C,i}$ are sampled directly from the Combined SSM model parameter distribution ${b}_{C,i} \sim N(0,{\lambda }_{C,i})$ and the resulting plausibilities are calculated. Since the plausibilities of generated shapes would be uniformly distributed between 0 and 1 if the shape generating mechanism is unbiased, the plausibilities are visualized and assessed using the Kolmogorov–Smirnov (“KS”) Test [53] for the hypothesis of non-uniformity.

## Results

### Subject 3D volume representations

**Fig. 3:**
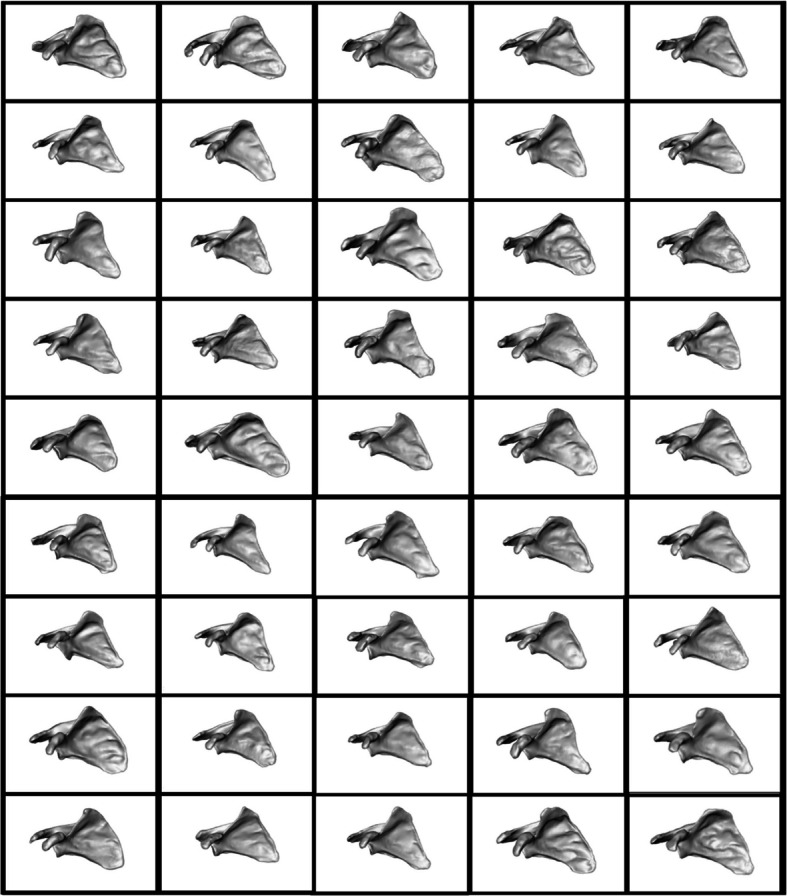
Scapular volumes input to ShapeWorks Studio for point correspondence

**Fig. 4:**
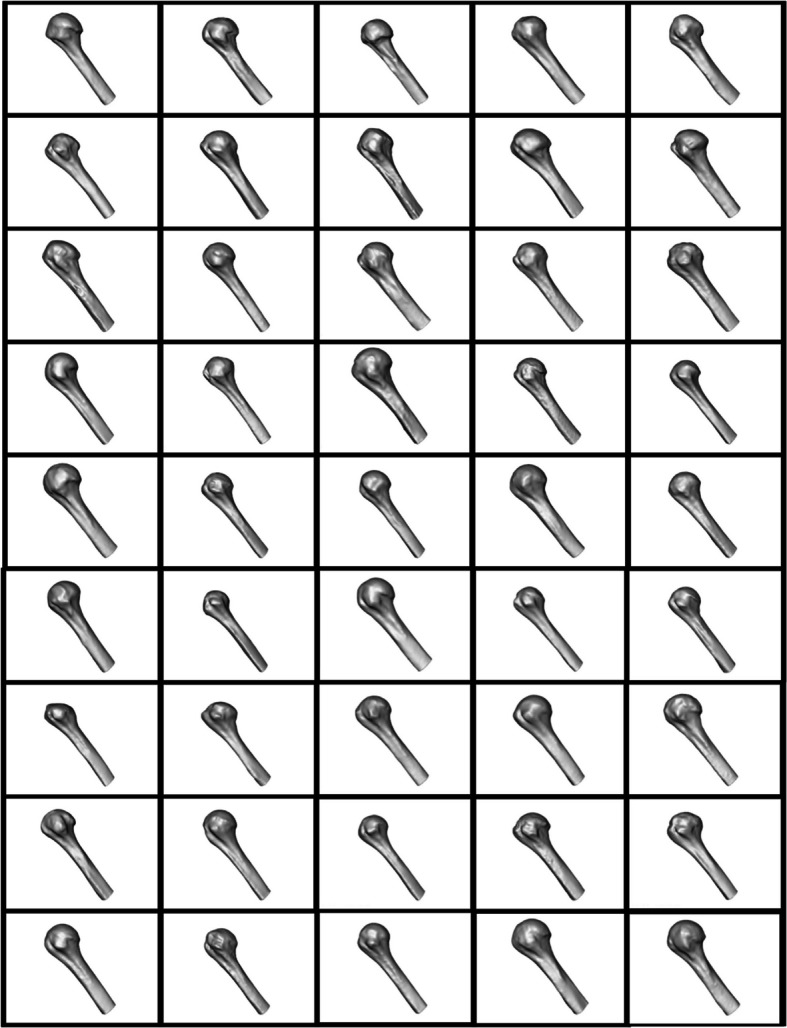
Proximal humeral volumes input to ShapeWorks studio for point correspondence

The 45 scapular and proximal-humeral samples resulting from segmentation, remeshing, and reflection are depicted in Figs. 3 and 4.

### Generated statistical shape models

**Fig. 5:**
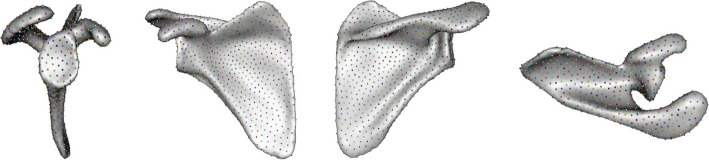
Scapula-only SSM training set average point cloud-lateral, anterior, posterior, and superior views

**Fig. 6:**
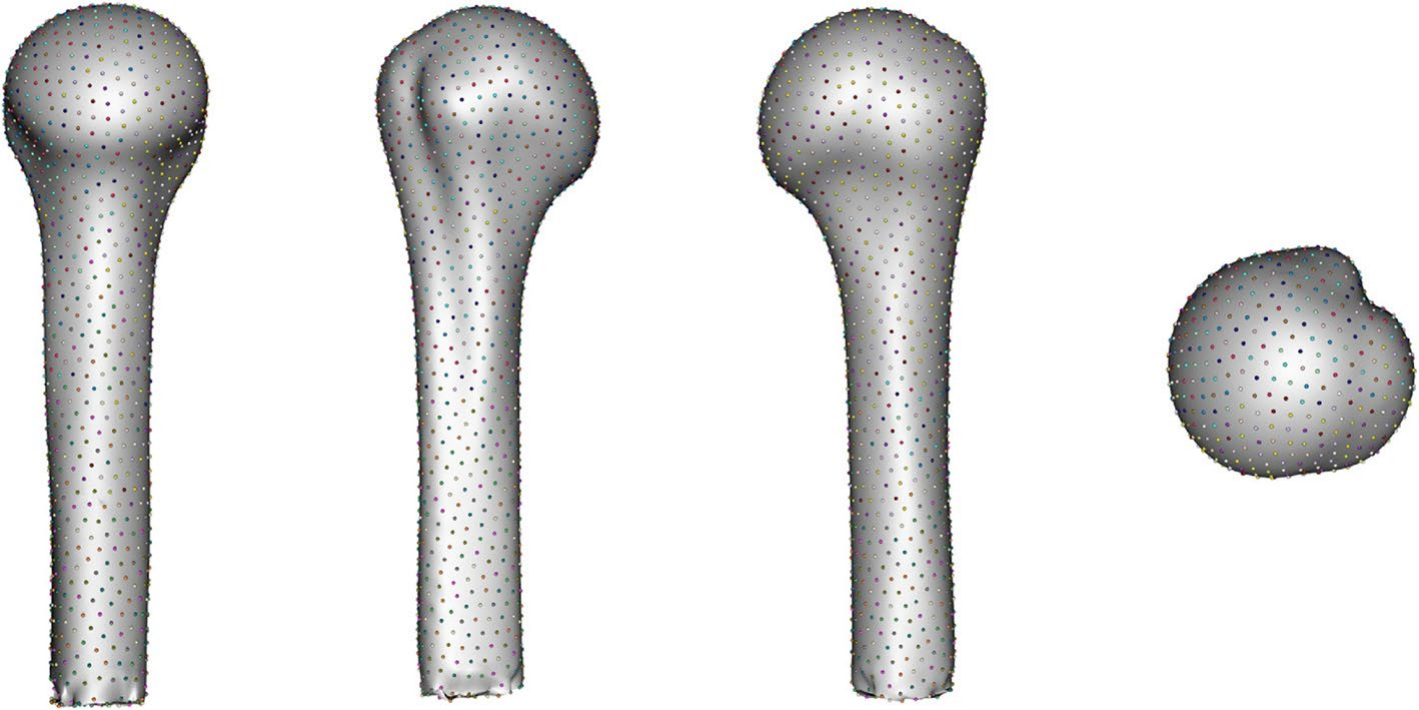
Proximal-humerus-only SSM training set average point cloud-medial, anterior, posterior, and superior views

**Fig. 7:**
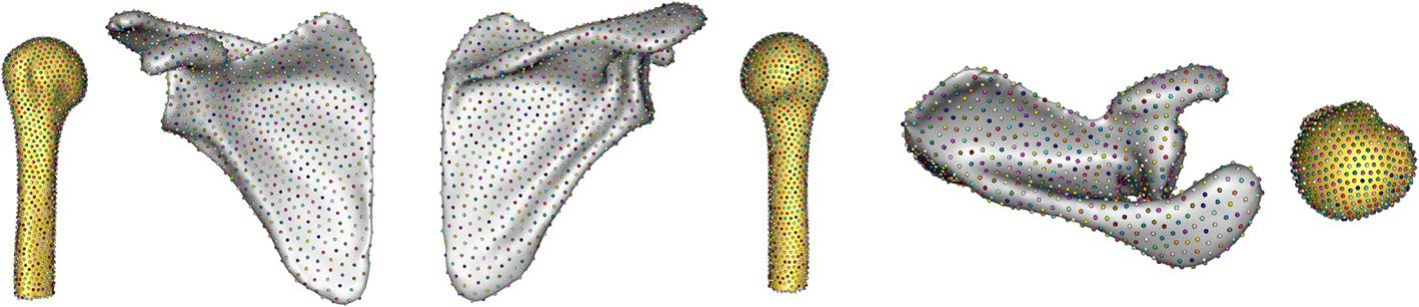
Combined two-body SSM training set average point cloud-anterior, posterior, and superior views

Visualisations of the training set averages $\overline{{\varvec{x}} }$ for the Scapula SSM (Fig. 5), Proximal-Humerus SSM (Fig. 6), and Combined SSM (Fig. 7), follow.

### Model descriptive ground truth accuracy

**Fig. 8:**
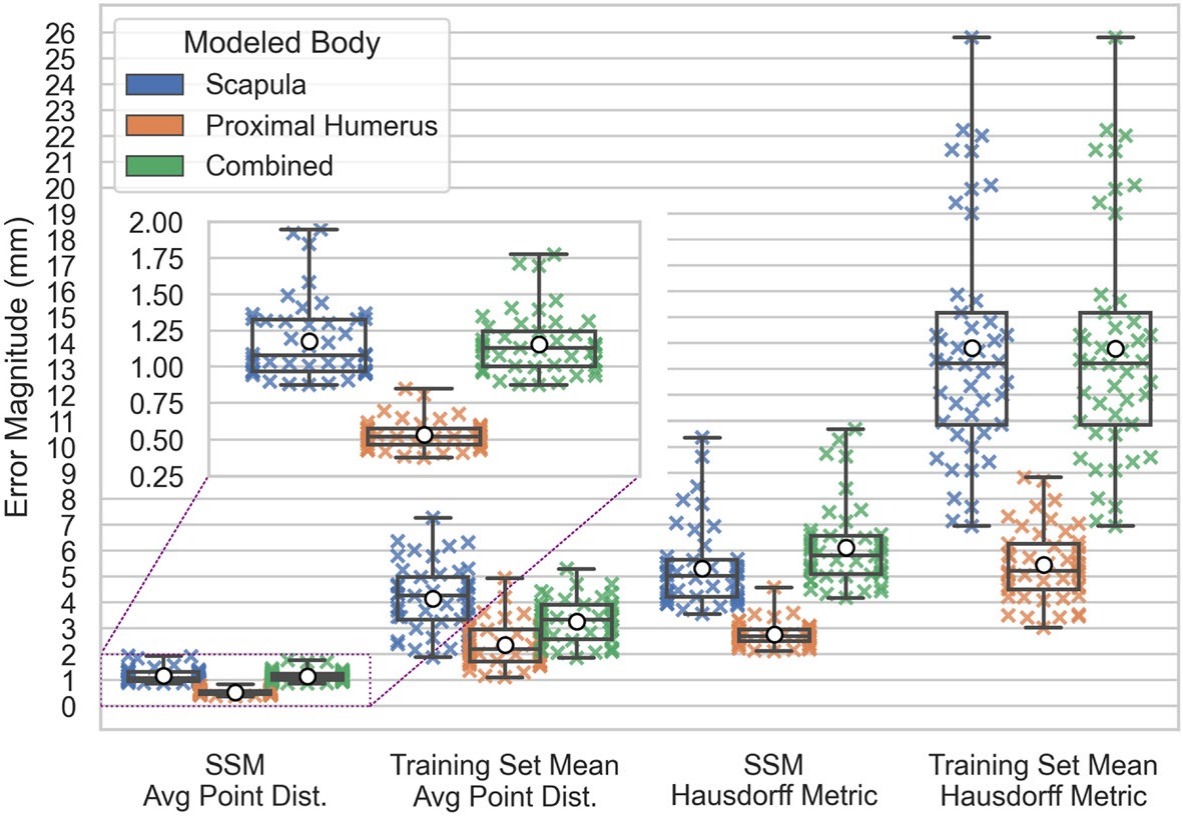
Leave-One-Out Cross-Validation Errors for SSMs and Training Set Means. X’s denote test observations, box plots denote observation quartiles and extremes, and white circles denote observation averages

The results of the leave-out-one cross-validation tests are plotted in Fig. 8. Ground Truth Accuracy is markedly improved when using an SSM compared to using the mean shape of the training set. The Scapula and Combined SSMs have consistently higher errors than the Proximal-Humerus SSM.

### Model generalisation ability: number of training samples

**Fig. 9:**
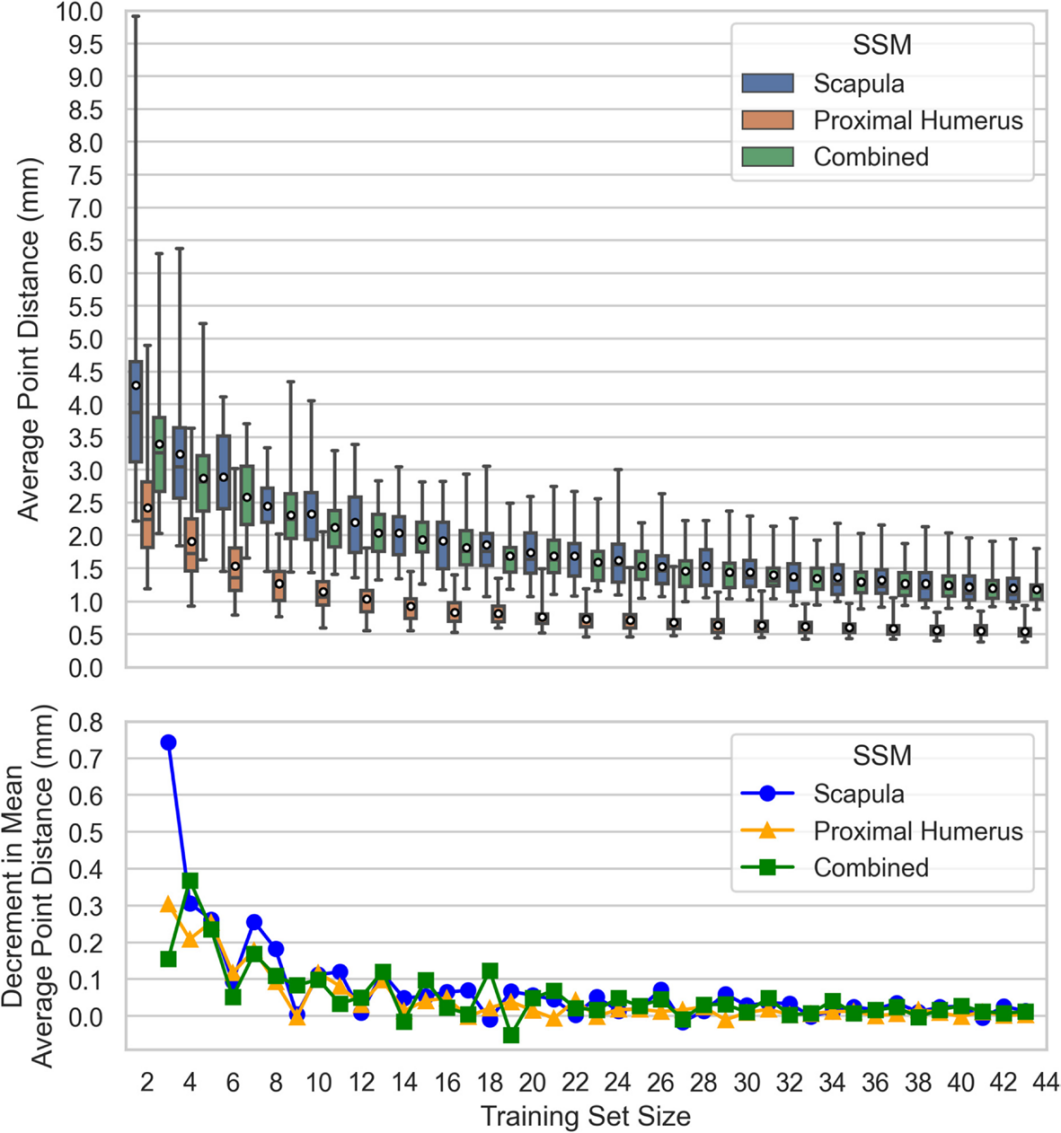
Generalisation Ability across Number of Training Samples for each SSM. (A) The distribution of distances between model outputs and the target shape point clouds (every odd Training Set Size shown), box plots denote observation quartiles and extremes and white circles denote distribution averages; and (B) The incremental decrease in mean average target shape point cloud distance for each additional training set sample

The *Generalisation* of each SSM across training set size is provided in Fig. 9. These results consist of error distributions with decreasing means and ranges plus a random error signal introduced by the random sampling with which the training sets were selected. The Combined SSM shows superior performance initially but converges with the Scapula SSM, whereas the Proximal-Humerus SSM consistently achieves the lowest point distances. The mean average point distances for all three SSMs exhibit similar convergence patterns as functions of training set size.

### Model generalisation ability: number of principal components

**Fig. 10:**
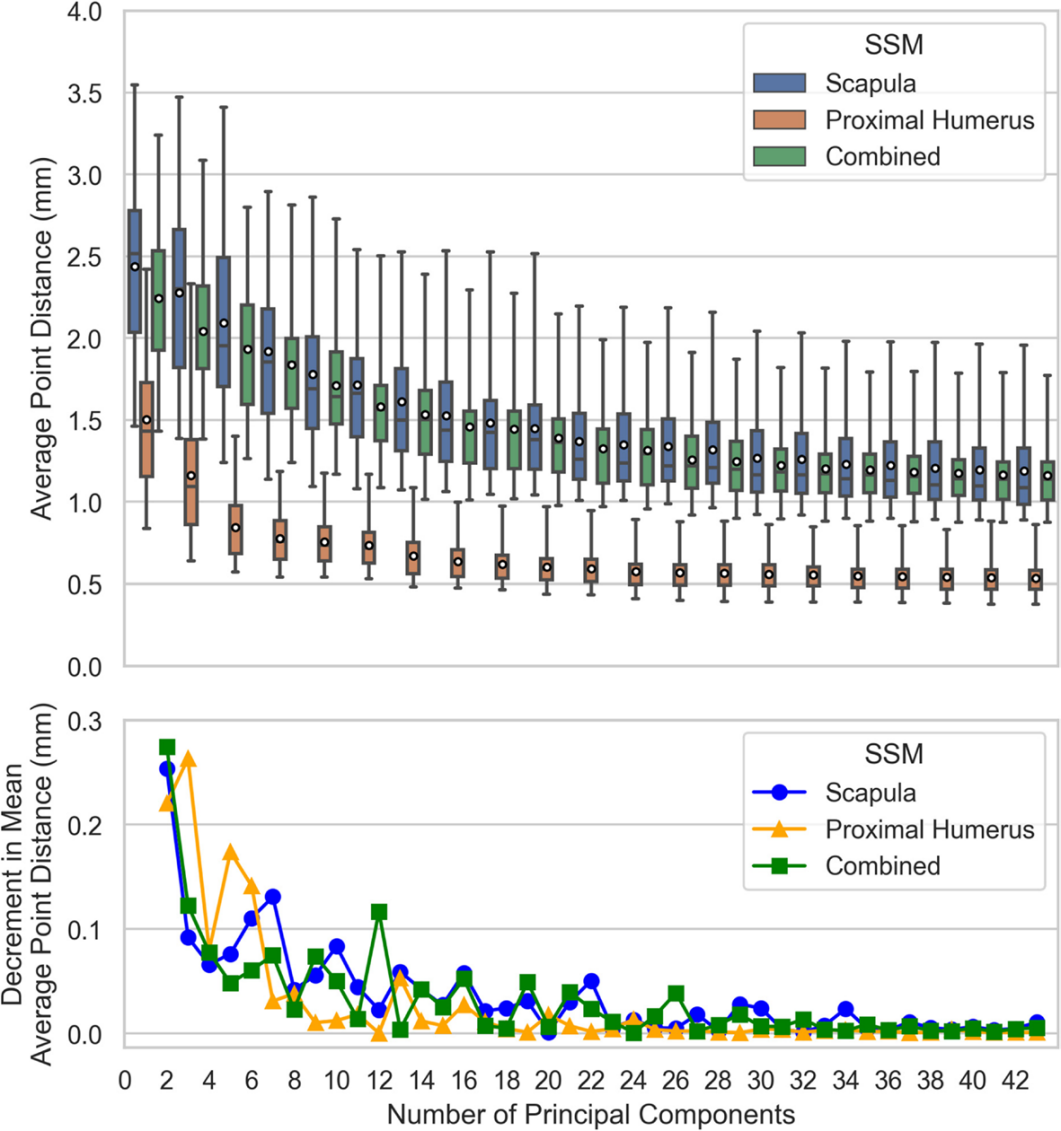
Generalisation Ability across Number of Principal Components for each SSM. (A) The distribution of distances between model outputs and the target shape point clouds (every odd Number of Principal Components shown), box plots denote observation quartiles and extremes and white circles denote distribution averages; and (B) The incremental decrease in mean average target shape point cloud distance for each additional Principal Component included in the SSM

The *Generalisation* of each SSM across the number of included principal components is provided in Fig. 10. Similar to the previous section, these results consist of error distributions with decreasing means and ranges. The Scapula and Combined SSM perform comparably, and the Proximal-Humerus SSM has consistently lower point distances. The mean average point distances for all three SSMs exhibit similar convergence patterns as functions of training set size, with the Proximal-Humerus SSM converging first, followed by the Scapula SSM, and finally the Combined SSM.

### Model specificity

**Fig. 11:**
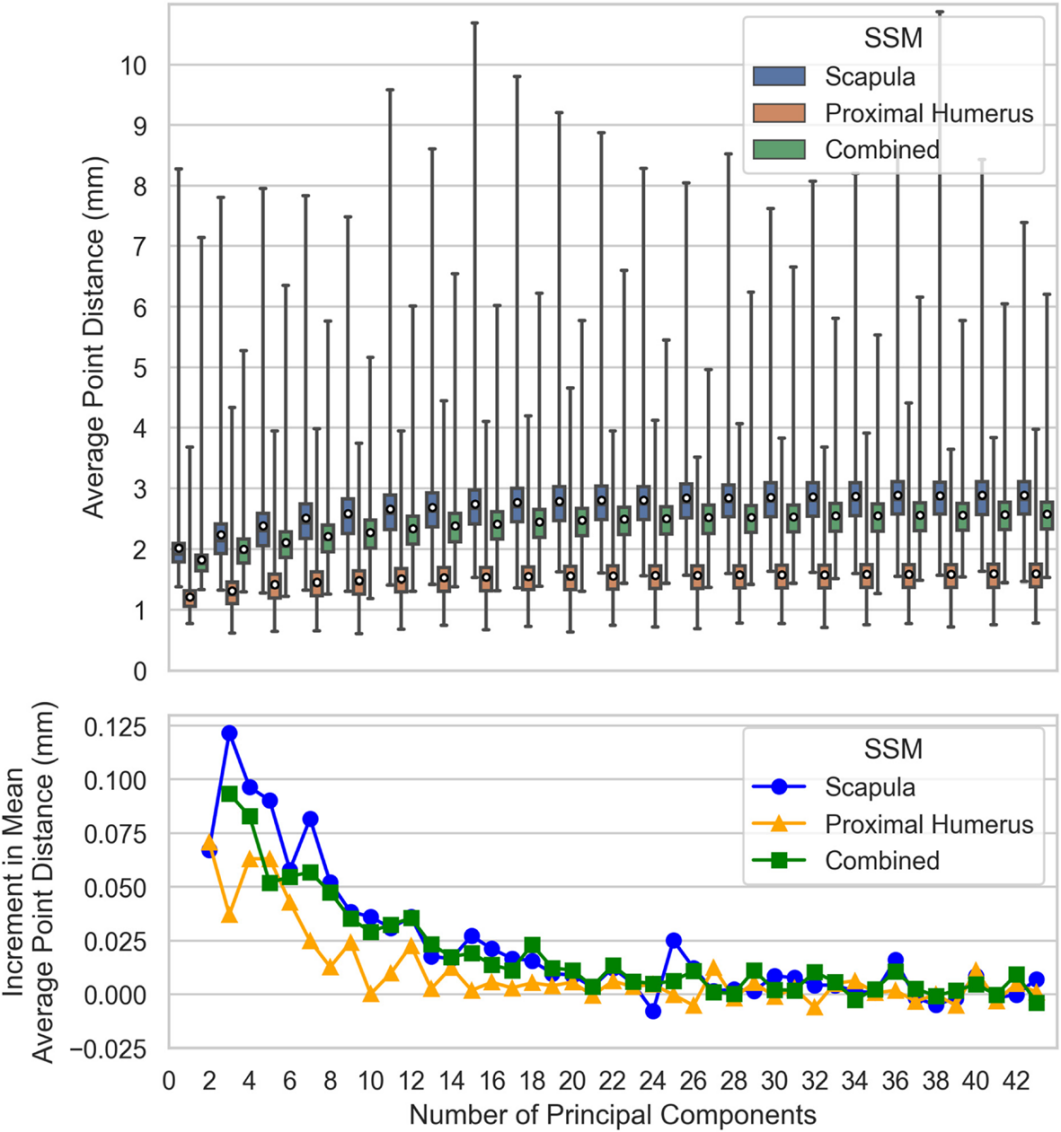
Specificity across Number of Principal Components for each SSM. (A) The distribution of distances between model outputs and the closest training set shape point clouds (every odd Number of Principal Components shown), box plots denote observation quartiles and extremes and white circles denote distribution averages; and (B) The incremental increase in mean average target shape point cloud distance for each additional Principal Component included in the SSM

The *Specificity* of each SSM across the number of included principal components, with $M=\text{10,000}$ randomly generated shape instances for each test, is provided in Fig. 11. The SSMs display first-order dynamics, with generated shapes initially closely resembling the training sets and gradually converging at greater average distances. The Combined SSM performs similarly to the Scapula SSM but with a reduced range, whereas the Proximal-Humerus SSM shows significantly lower mean average point distances and a narrower distance range compared to the other two.

### Model compactness

**Fig. 12:**
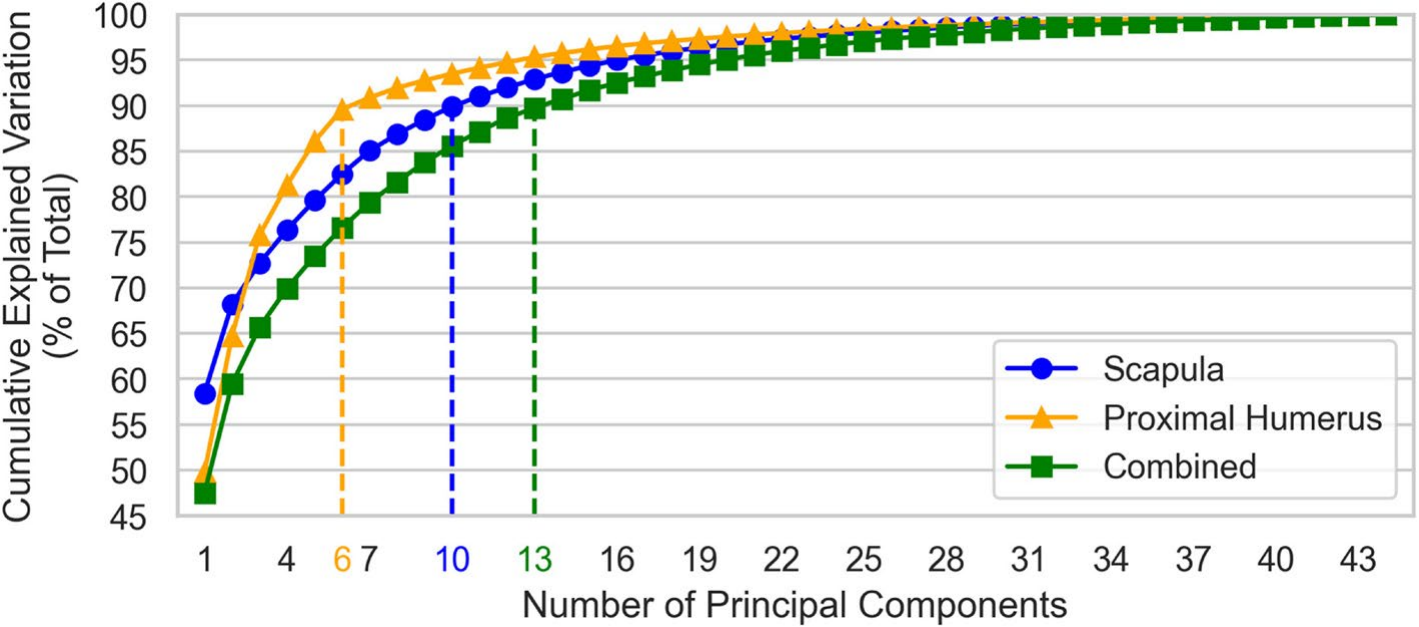
Compactness of each SSM. The number of principal components required to capture at least 90% of the total data set variation for each shape is designated by the vertical dashed lines and color-coordinated x-axis tick labels

The total training set variation explained by number principal components, for each SSM, is provided in Fig. 12. The Proximal-Humerus SSM is the most compact, followed by the Scapula SSM, with the Combined SSM being the least compact.

### SSM modes of variation

**Fig. 13:**
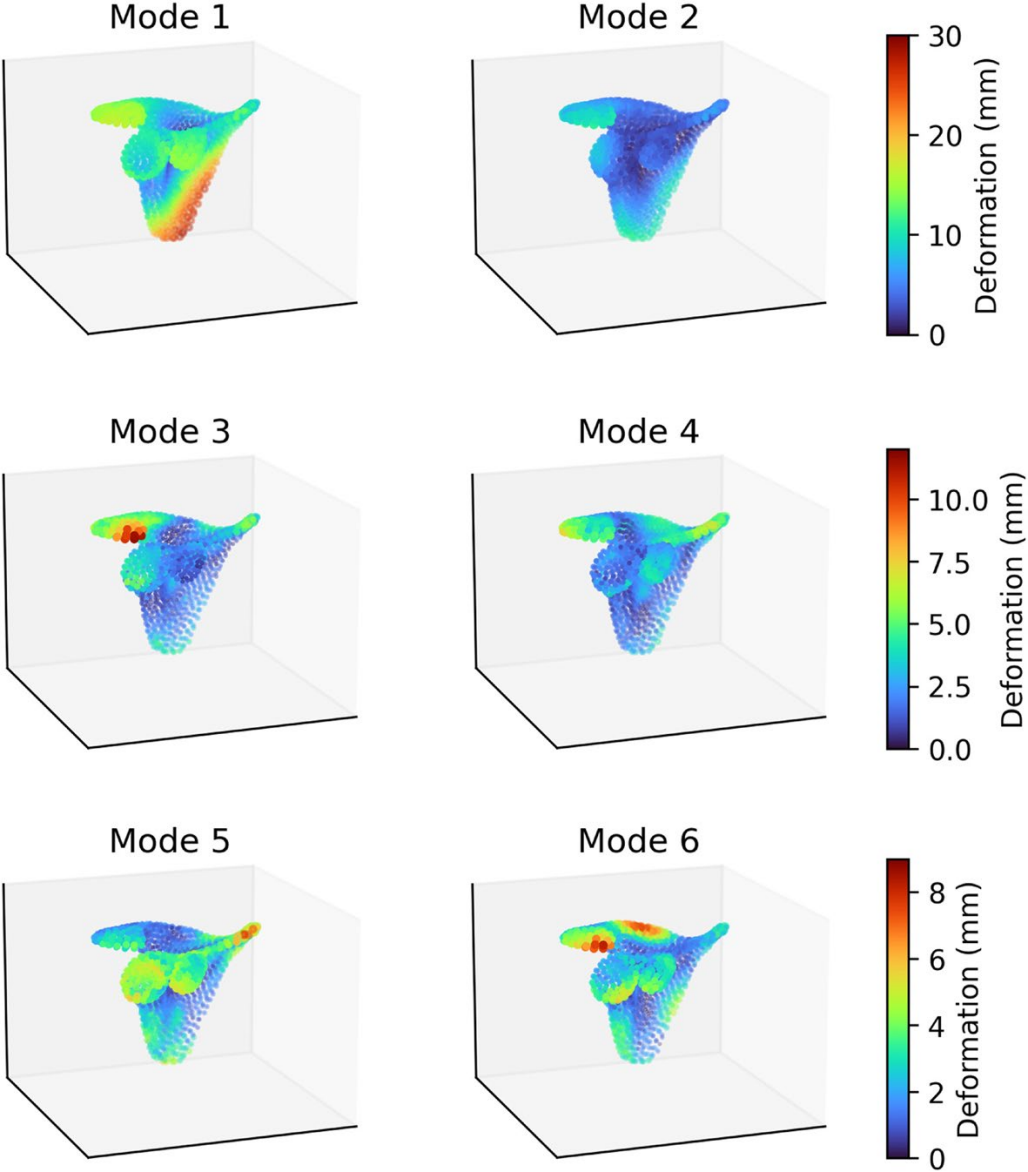
Primary 6 Modes of Variation of the Scapula SSM, Lateral Oblique View. The extent of point displacement along the mode of variation is encoded in marker size (small to large) and colour (blue through yellow to red)

**Fig. 14:**
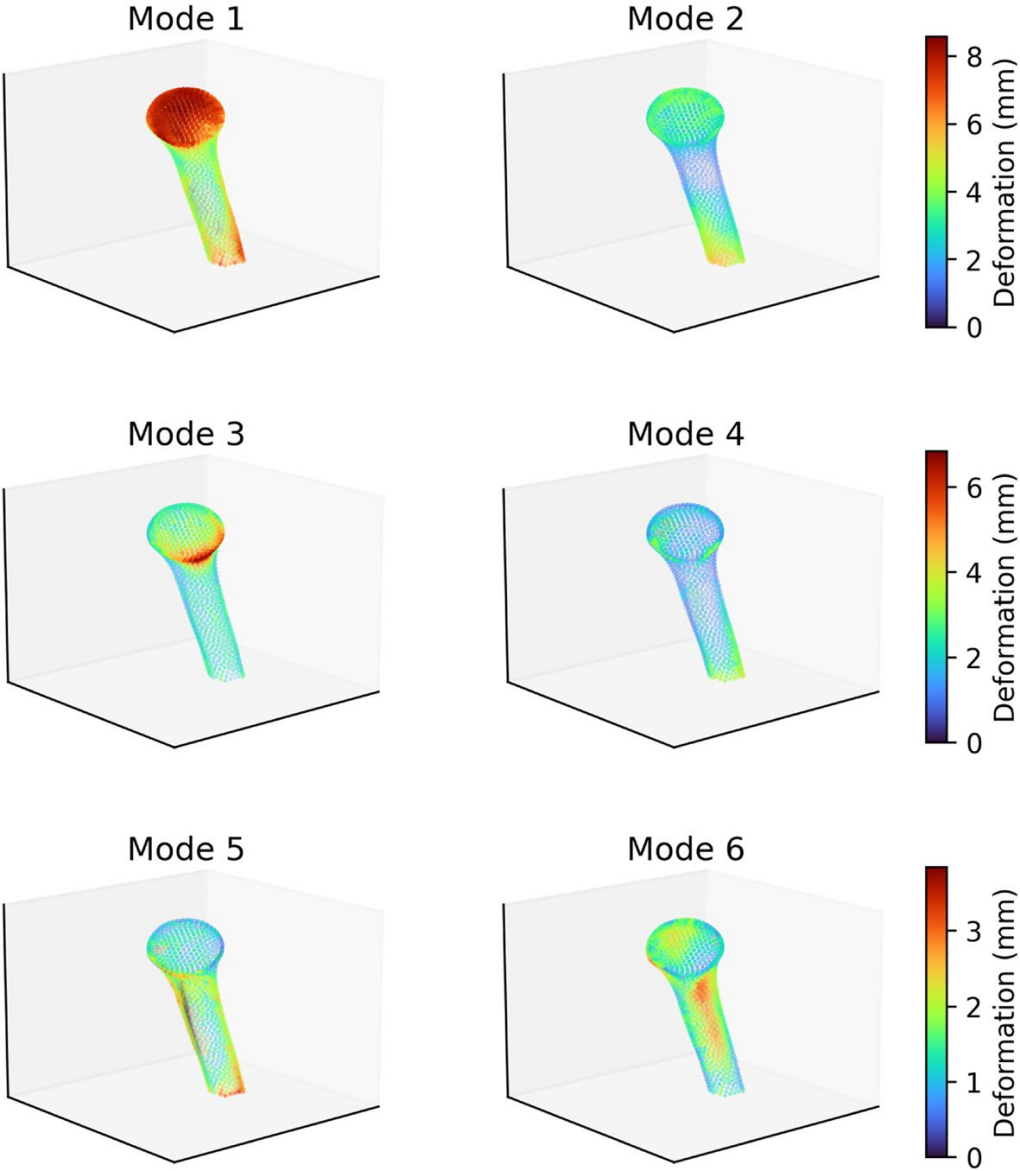
Primary 6 Modes of Variation of the Proximal-Humerus SSM, Medial View. The extent of point displacement along the mode of variation is encoded in marker size (small to large) and colour (blue through yellow to red)

**Fig. 15:**
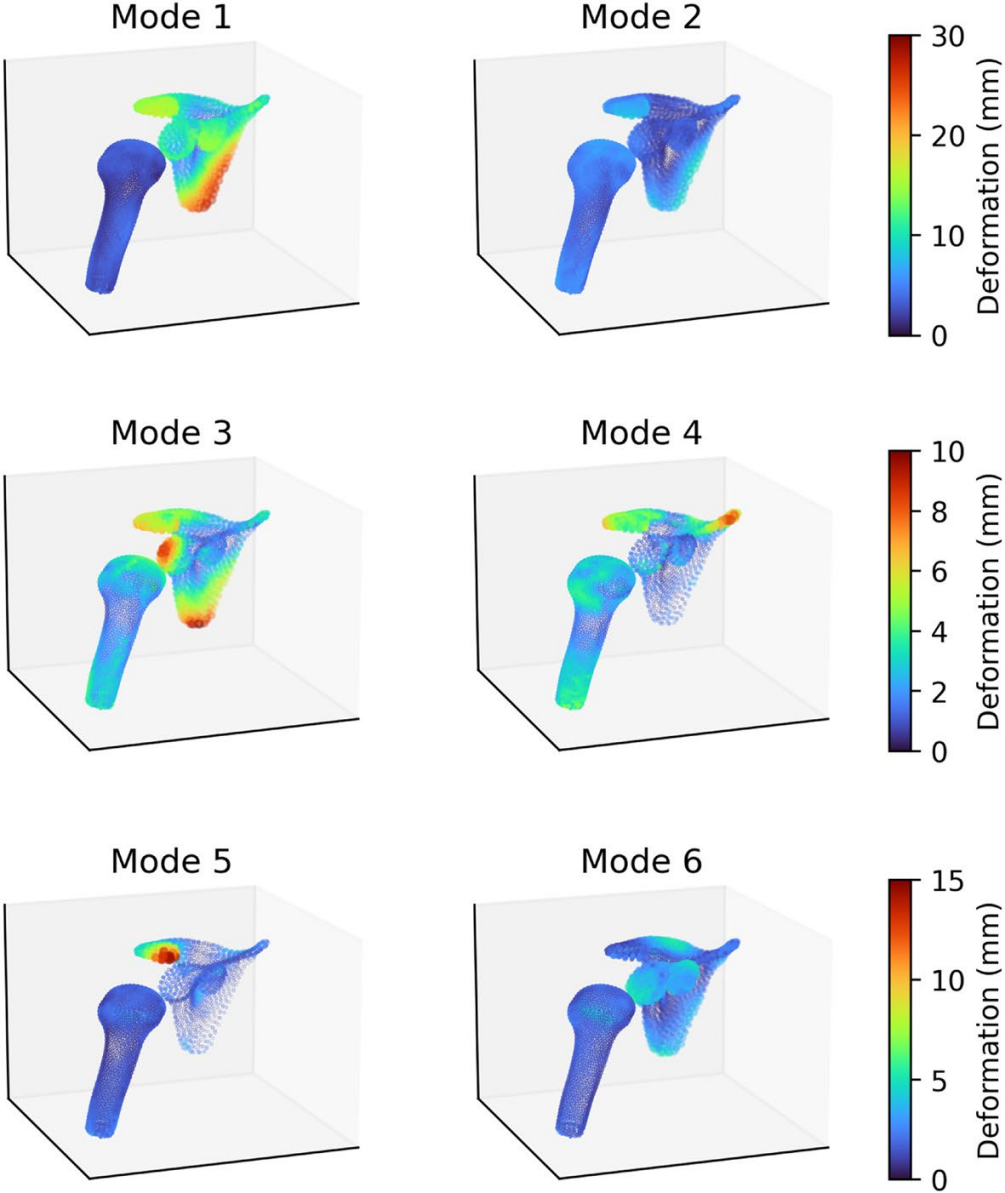
Primary 6 Modes of Variation of the Combined SSM, Lateral Oblique View. The extent of point displacement along the mode of variation is encoded in marker size (small to large) and colour (blue through yellow to red)

The shape variations contributed by the first 6 principal components (“modes”) of the Scapula SSM are depicted in Fig. 13 by visualising the extent of point displacement caused by adding three standard deviations along each mode of variation, $3\sqrt{{\lambda }_{i}}{ \boldsymbol{\varphi }}_{i}$, to the training set average point cloud $\overline{{\varvec{x}} }$. The same is depicted for the Proximal-Humerus SSM in Fig. 14, and for the Combined SSM in Fig. 15.

The range of radiographic morphologic characteristics of scapular anatomy [9] observed when varying the Scapula SSM across three standard deviations in the first three modes is listed in Table 6. The same is depicted for the Proximal-Humerus SSM [31–34] in Table 7, and for the Combined SSM in Table 8.

**Table 6:** Morphologic characteristics across scapula SSM modes

Dimension	Mean	Mode 1 ± 3SD Range (Min—Max)	Mode 2 ± 3SD Range (Min—Max)	Mode 3 ± 3SD Range (Min—Max)
Scapula Height (mm) [9]	154	62.4 (124—186)	22.3 (143—166)	12.9 (148—161)
Scapula Width (mm) [9]	104	40.6 (83.2—124)	11.7 (97.8—109)	6.83 (100—107)
Scapula Aspect Ratio (Width/Height)	0.672	0.008 (0.666—0.674)	0.174 (0.591—0.764)	0.101 (0.623—0.724)
Glenoid Height (mm) [9]	39.6	19.0 (30.1–49.0)	6.94 (36.1–43.0)	1.70 (38.7—40.4)
Glenoid Width (mm) [9]	29.1	16.2 (21.0—37.3)	2.74 (27.8—30.5)	0.388 (29.0—29.3)
Acromion Length (mm) [9]	45.3	25.8 (32.5—58.3)	19.2 (37.1—56.3)	4.11 (44.1—48.2)
Lateral Acromion to Glenoid Center (mm) [9]	28.5	11.3 (22.9—34.1)	8.01 (24.3—32.3)	2.63 (27.2—29.9)
Coracoid Tip to Glenoid Center (mm) [9]	16.3	5.77 (13.4—19.2)	6.74 (13.0—19.7)	5.27 (13.6—18.9)
Posterior-Inferior Acromion to Glenoid Center (mm) [9]	40.1	22.4 (29.1—51.5)	0.556 (39.6—40.1)	12.8 (33.7—46.5)
Superior-Anterior Acromion to Glenoid Center (mm) [9]	4.94	0.658 (4.68—5.34)	13.1 (4.94–18.0)	11.3 (4.94—16.3)
Fulcrum Axis (°) [9]	94.1	0.255 (94—94.2)	5.65 (91.4—97.1)	1.02 (93.5—94.5)
Glenoid Inclination Angle (°) [9]	97.6	2.88 (95.8—98.7)	1.42 (97.2—98.7)	0.305 (97.5—97.8)
Glenoid Version Angle (°) [9]	94.4	5.33 (90.9—96.3)	24.1 (81.5—106)	9.21 (89.7—98.9)
Acromial Tilt Angle (°) [9]	31.8	1.64 (30.7—32.3)	13.0 (27.7—40.7)	10.7 (27.0—37.8)
Critical Shoulder Angle (°) [9]	29.4	0.888 (28.8—29.7)	3.85 (27.8—31.7)	2.29 (28.3—30.6)
Superior-Inferior Glenoid-Acromion Angle (°) [9]	52.9	2.80 (52.0—54.8)	27.2 (36.4—63.7)	19.5 (43.1—62.6)
Superior-Inferior Glenoid-Acromion-Coracoid Angle (°) [9]	95.3	1.17 (94.5—95.7)	14.3 (88.6—103)	8.75 (90.4—99.2)

**Table 7:** Morphologic characteristics across proximal-humerus SSM modes

Dimension	Mean	Mode 1 ± 3SD Range (Min—Max)	Mode 2 ± 3SD Range (Min—Max)	Mode 3 ± 3SD Range (Min—Max)
Proximal Humeral Length (mm)	152	25.3 (140—165)	1.90 (152—154)	7.36 (149—156)
Humeral Shaft Diameter (mm) [12]	22.2	13.0 (15.8—28.7)	1.74 (21.3—23.1)	2.51 (21.0—23.5)
Humeral Head Radius of Rotation (mm) [31]	22.9	9.43 (18—27.4)	0.636 (22.7—23.4)	3.71 (21.7—25.4)
Humeral Head Inclination Angle (°) [32]	139	35.6 (105—140)	1.47 (138—139)	23.1 (116—139)
Humeral Head Medial Offset (mm) [33]	1.75	2.96 (0.175—3.14)	0.672 (1.35—2.02)	2.71 (0.831—3.54)
Humerus Greater Tuberosity Angle (°) [34]	67.5	1.16 (66.3—67.5)	5.45 (63.1—68.5)	21.9 (45.5—67.5)

**Table 8:** Morphologic characteristics across combined SSM modes

Dimension	Mean	Mode 1 ± 3SD Range (Min—Max)	Mode 2 ± 3SD Range (Min—Max)	Mode 3 ± 3SD Range (Min—Max)
Scapula Height (mm) [9]	154	62.8 (123—186)	7.65 (150—158)	19.9 (144—164)
Scapula Width (mm) [9]	104	40.0 (83.6—124)	12.1 (97.5—110)	8.64 (99.3—108)
Scapula Aspect Ratio (Width/Height)	0.672	0.0138 (0.664—0.678)	0.112 (0.617—0.729)	0.143 (0.604—0.747)
Glenoid Height (mm) [9]	39.6	19.1 (30.0—49.1)	2.84 (38.1—41.0)	6.42 (36.3—42.7)
Glenoid Width (mm) [9]	29.1	16.4 (20.9—37.3)	1.80 (28.2—30.0)	0.341 (29.0—29.4)
Acromion Length (mm) [9]	45.3	26.2 (32.3—58.5)	9.73 (41.2—51.0)	15.0 (38.4—53.4)
Lateral Acromion to Glenoid Center (mm) [9]	28.5	11.3 (22.9—34.2)	2.26 (27.2—29.5)	8.02 (24.3—32.4)
Coracoid Tip to Glenoid Center (mm) [9]	16.3	5.96 (13.3—19.3)	3.85 (14.4—18.2)	5.42 (13.6—19.0)
Posterior-Inferior Acromion to Glenoid Center (mm) [9]	40.1	22.1 (29.2—51.3)	3.33 (38.2—41.6)	3.47 (38.2—41.7)
Superior-Anterior Acromion to Glenoid Center (mm) [9]	4.94	0.441 (4.79—5.23)	9.80 (4.41—14.2)	9.85 (3.26—13.1)
Fulcrum Axis (°) [9]	94.1	0.209 (94.0—94.2)	5.74 (91.3—97.1)	0.753 (93.8—94.5)
Glenoid Inclination Angle (°) [9]	97.6	2.70 (95.9—98.6)	2.02 (96.8—98.8)	0.439 (97.5—98)
Glenoid Version Angle (°) [9]	94.4	5.26 (91.0—96.2)	9.41 (89.5—98.9)	25.0 (81.5—106)
Acromial Tilt Angle (°) [9]	31.8	1.29 (30.9—32.2)	7.04 (29.1—36.2)	11.1 (27.6—38.7)
Critical Shoulder Angle (°) [9]	29.4	0.492 (29.0—29.5)	4.93 (27.1—32.0)	0.978 (29—29.9)
Superior-Inferior Glenoid-Acromion Angle (°) [9]	52.9	1.48 (52.4—53.9)	19.8 (42—61.8)	18.0 (42.4—60.4)
Superior-Inferior Glenoid-Acromion-Coracoid Angle (°) [9]	95.3	0.190 (95.2—95.3)	12.8 (89.0—102)	6.97 (92.0—99.0)
Proximal Humeral Length (mm)	152	10.3 (148—158)	20.0 (143—163)	11.2 (147—158)
Humeral Shaft Diameter (mm) [12]	22.2	7.84 (18.3—26.1)	10.3 (17.1—27.4)	2.39 (21—23.4)
Humeral Head Radius of Rotation (mm) [31]	22.9	3.99 (21.3—25.3)	7.78 (19—26.8)	2.06 (21.7—23.8)
Humeral Head Inclination Angle (°) [32]	139	40.4 (98.8—139)	10.8 (131—142)	29.0 (110—139)
Humeral Head Medial Offset (mm) [33]	1.75	1.90 (0.167—2.07)	1.54 (0.925—2.46)	4.18 (0.906—5.09)
Humerus Greater Tuberosity Angle (°) [34]	67.5	8.96 (64.0—73.0)	5.14 (62.5—67.6)	13.5 (53.9—67.5)

### Quantification of coupled scapulohumeral variation

**Fig. 16:**
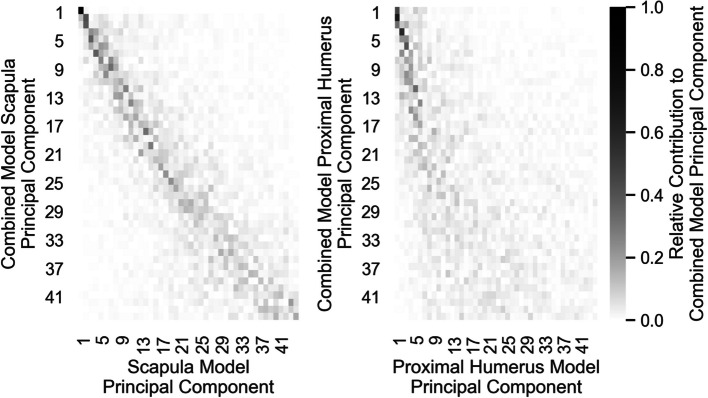
Decomposition of the modes of variation of the combined SSM into single-body SSM principal components

The decomposition of the Combined SSM scapular and proximal-humeral eigenvector components into relative magnitude of Scapula SSM and Proximal-Humerus SSM eigenvector contributions, ${{\varvec{A}}}_{S}$ and ${{\varvec{A}}}_{H}$, is provided in Fig. 16. The scapular component decomposition of the Combined SSM more closely approximates an identity matrix compared to the proximal humeral component decomposition. The later Combined SSM component decompositions become progressively diffuse, with a greater number of constituents each contributing less.

**Fig. 17:**
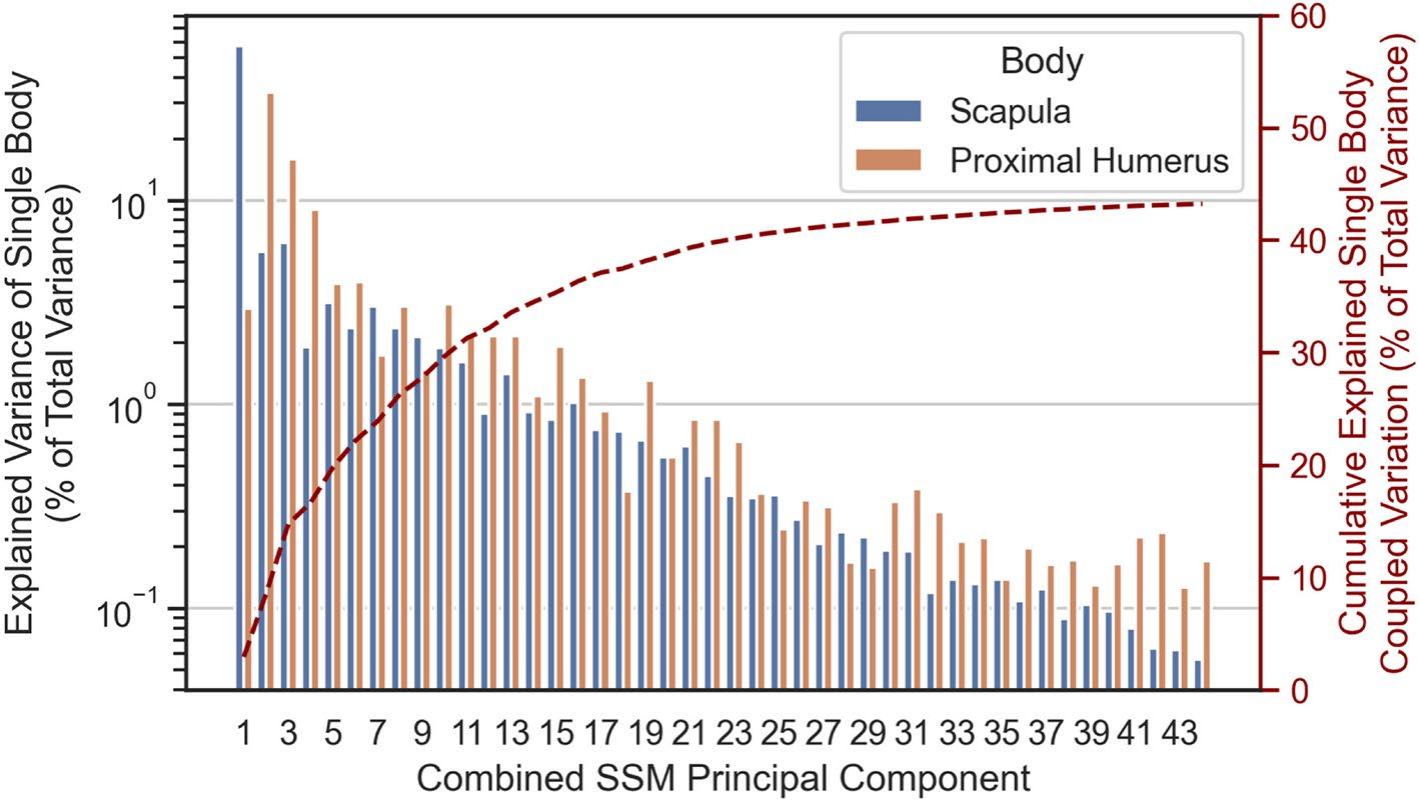
Single-body variation and cumulative coupled variation in each combined SSM principal component

The proportion of single-body shape variation captured in each Combined SSM Principal Component, ${{\varvec{v}}}_{C,S,j}$ and ${{\varvec{v}}}_{C,H,j}$ are provided in Fig. 17, along with the cumulative sum of the coupled variation of the two bodies that jointly occurs along Combined SSM Principal Components, ${{\varvec{v}}}_{J,j}$.

### Prediction of missing counterpart

**Fig. 18:**
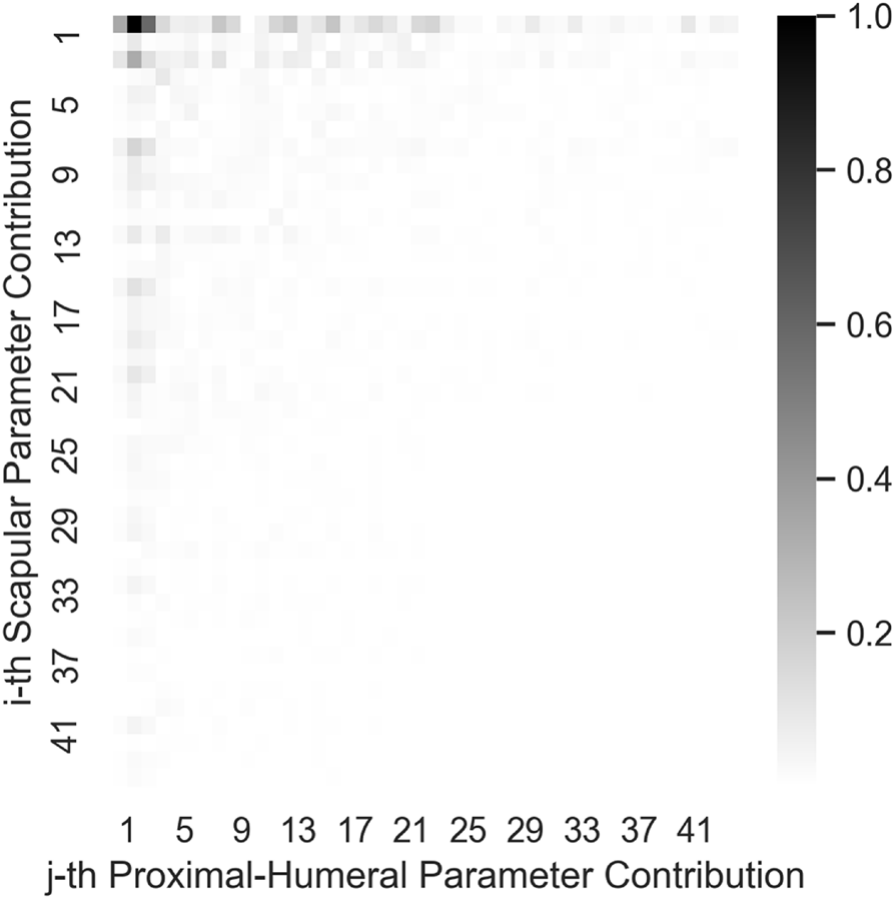
Relative magnitude of covariance between combined SSM model parameter single-body contribution decompositions

The relative magnitude of elements of the covariance matrix of the scapular and proximal-humeral contributions to Combined SSM fitted model parameters across the study data set, ${{\varvec{A}}}_{B}$, is visualized with the heatmap in Fig. 18; the matrix is sparse with significant magnitudes only occurring in the earlier rows and columns.

**Fig. 19:**
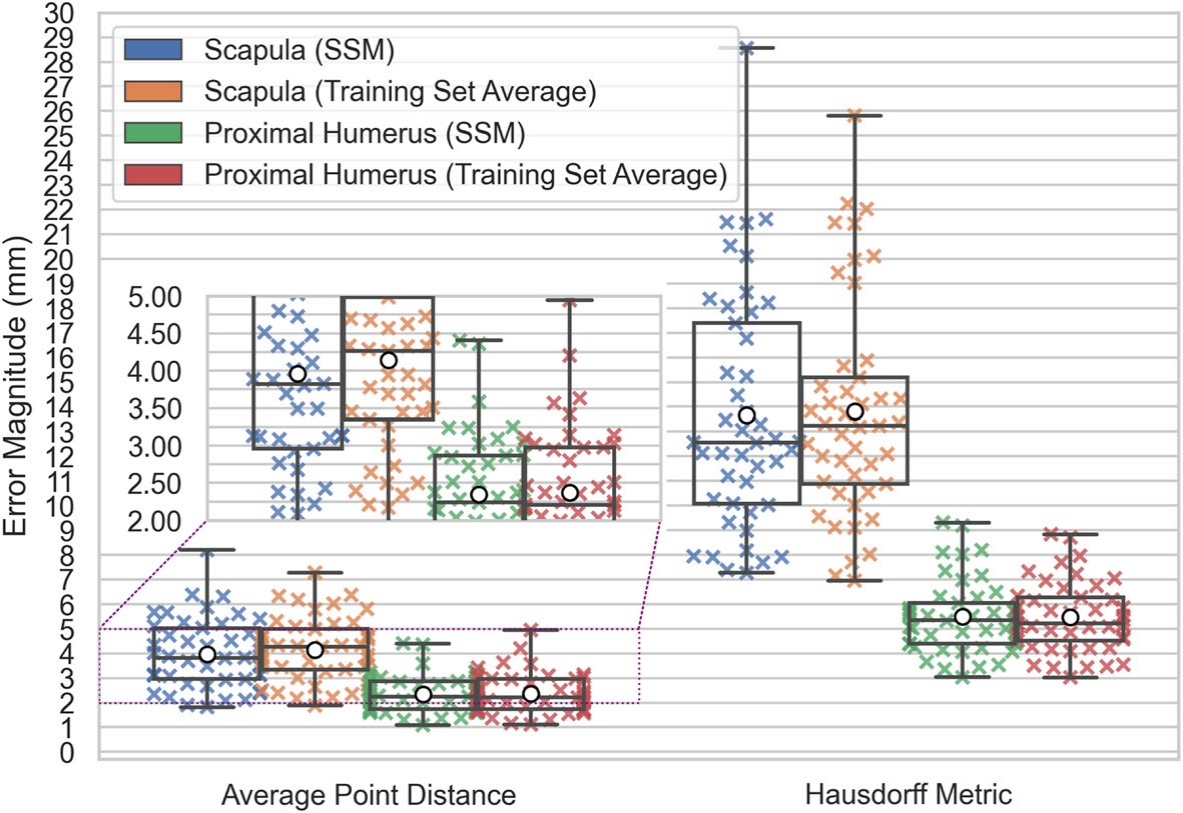
Leave-One-Out Cross-Validation for Missing Scapulae and Proximal Humeri. X’s denote test observations, box plots denote observation quartiles and extremes, and white circles denote observation averages

The results of the missing body prediction test are plotted in Fig. 19. The use of the SSM lowers the median Scapula prediction error magnitude but increases the worst-case errors, compared to the training set average. The Proximal-Humerus SSM decreases the error range compared to the training set average.

### De Novogeneration of synthetic populations

**Fig. 20:**
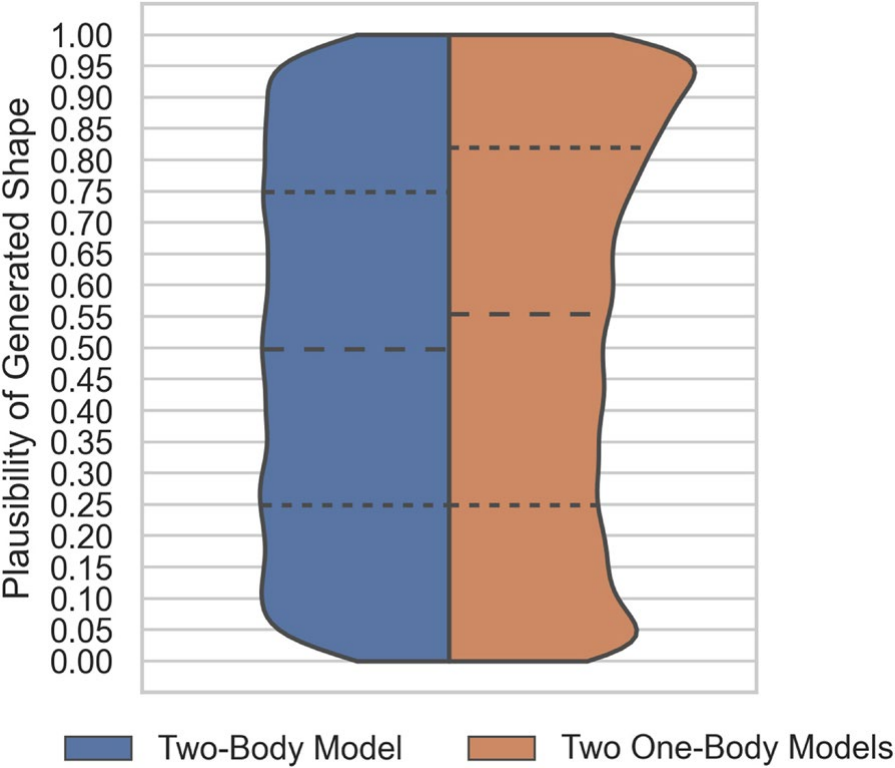
Combined SSM and One-Body SSM Model Generated Shape Plausibility. The width of the shaded area depicts the density of shape instances with that plausibility, and the dashed lines represent plausibility quartiles for each sample group

The distribution of plausibilities (*p*-values) resulting from the shapes generated by the Combined SSM compared to the two independent one-body SSMs is graphed in Fig. 20. The plausibilities generated by the two independent one-body SSMs exhibit two distinct peaks, one at high plausibility and the other at low plausibility.

The KS Test for non-uniformity of the shape plausibilities (where *p* < .05 suggests a sampling from a uniform distribution) results in *p* $< .001$ for the shapes generated by the Combined SSM and *p* = $.585$ for the shapes generated from the two independent one-body SSMs.

## Discussion

The analysis in the following subsections validates the independent single-body SSMs, as their performance aligns with previously reported models. Although no prior two-body SSM exists for direct comparison, the Combined SSM exhibits intuitive, explainable behavior relative to the independent single-body SSMs. Similarly, the primary modes of variation identified by the single-body SSMs are consistent with those documented in the literature. Notably, Mode 3 of the Combined SSM captures the pathological variation of the joint by encoding the covariation between glenoid and humeral head erosion. In fact, the Combined SSM demonstrates that 43.2% of the total scapular and proximal-humeral shape variation occurs in tandem. This coupled variation enables the Combined SSM to improve missing counterpart predictions, achieving over a 10% reduction in median error and a more than 9% reduction in error interquartile range for missing scapulae and proximal humeri, respectively. Conversely, the use of two independent single-body models for de novo synthetic population generation introduces bias, producing both implausible (mismatched) shape pairs and insufficient diversity within the generated population. We envision the novel combined SSM contributing to surgical decision-making and planning by: enhancing evidence-based selection of optimal RTSA prosthesis configuration and placement, providing a new quantitative measure of the severity of arthropathy, guiding complex reconstruction in the setting of comminuted scapular or humeral fractures, and automating the radiographic identification of glenohumeral arthropathy.

### Subject 3D volume representations

Notable variability is observed in the extent of glenoid erosion among the scapulae and head erosion in the humeri. These findings align with the anticipated heterogeneity of the clinical population, which comprises shoulder arthritis patients undergoing RTSA. The varying degrees of erosion reflect the diversity in disease progression and severity across the study cohort, reinforcing the importance of capturing such anatomical variation for accurate statistical shape modeling.

### Generated statistical shape models

The mean shapes of both the scapula and humerus demonstrate acceptable correspondence point dispersion, effectively capturing the typical morphology of bony anatomy. This uniform distribution of points ensures an accurate representation of shape variations within the study population. There are no apparent incongruities in the point clouds, which would manifest as sharp disruptions or irregularities in the otherwise smooth 3D mesh formed between the points. The absence of such inconsistencies is suggestive of successful segmentation and correspondence optimization processes.

### Model descriptive ground truth accuracy

The Hausdorff metric is consistently greater than the average point distance across all cases, as expected since it serves as a worst-case distance measure. This metric highlights the outermost variations in shape representation, complementing the average point distance for assessing model accuracy.

All three SSMs significantly outperform the Training Set Mean predictions in terms of both error central tendency and dispersion. For example, the Combined SSM achieves an average point distance with median 1.13 mm (IQR 0.239 mm), compared to the Training Set Mean, which exhibits a median of 3.34 mm (IQR 1.32 mm). The difference in average point distance distributions between the SSM and the Training Set Mean is a direct measure of the descriptive benefit of the shape variation captured by the principal components of the SSMs.

The average point distance errors and Hausdorff metrics observed for the Scapula and Proximal-Humerus SSMs are within the range reported in the literature (Scapula mean RMS errors reported between 1.7 and 2.5 mm, maximum Hausdorff metrics reported between 5.5 and 18 mm; Humerus mean RMS errors reported between 0.48 and 2.1 mm, maximum Hausdorff Metrics reported between 4 and 5.5 mm) [11–15, 29]. The Proximal-Humerus SSM yields reduced errors compared to the Scapula SSM as expected given the higher complexity of scapular morphology. The Proximal-Humerus SSM achieves a Hausdorff metric distribution with median 2.71 mm (IQR 0.452 mm), while the Scapula SSM distribution has median 5.05 mm (IQR 1.42 mm).

The Combined SSM performs comparably to the Scapula SSM but demonstrates slightly higher central tendency errors with reduced dispersion. For example, the Combined SSM has an average point distance distribution with median 1.13 mm (IQR 0.239 mm), whereas the Scapula SSM achieves an error distribution with median 1.08 mm (IQR 0.359 mm).

Interestingly, an alternative and simpler *independent* two-body shape model, defined by the concatenation of the two independent single-body models, would yield error metrics that are averages of the two constituent model results, weighted by the number of points in their respective point clouds. In this case, the *independent* two-body model achieves an average point distance distribution with a median of 0.861 mm (IQR 0.553 mm). This outcome arises directly from the significant disparity in shape complexity between the scapula and proximal humerus, as well as the reduced constraints of the independent model, which does not need to account for coupled variations between the two bodies. In descriptive applications where the shapes of both bones are already provided, the increased complexity of the Combined SSM offers no additional value, as the coupled shape variations are intrinsic to the presented bodies and need not be predicted.

### Model generalisation ability: number of training samples

As was the case for Descriptive *Ground Truth Accuracy*, the Proximal-Humerus SSM outperforms the Scapula SSM in terms of absolute error by virtue of the simpler bone shape being modelled. However, the single-body SSMs perform similarly in terms of Generalization Ability by samples in reaching a decrease in mean average point distance of less than 0.1 mm per additional training set sample after only 10 inputs (Proximal-Humerus SSM) and 11 inputs (Scapula SSM). The convergence of Scapular SSM Generalisation across the number of training samples has previously been reported to occur within 23 inputs [9]; there is no published Generalisation ability data for proximal humerus or combined two-body models for comparison purposes. The Combined SSM results are more sensitive to random variation and reach the same diminishing return per additional sample after 18 inputs. At lower training set sizes, where the description accuracy disparity between the Proximal Humerus and Scapula is widest, the Combined SSM outperforms the Scapula SSM.

### Model generalisation ability: number of principal components

Generalisation Ability when varying the number of principal components included in each SSM is similar to that when varying the number of training samples except for the increased efficiency when using all training samples and varying the included number of modes. Also note that the Combined SSM performance moves in tandem with that of the Scapula SSM, rather than converging with the Scapula SSM, as was the case for Generalisation Ability when varying the number of training samples. In this case the Proximal-Humeral SSM reaches diminishing returns with less than 0.1 mm decrease in average point distance mean after 6 modes, the Scapula SSM after 7 modes, and the Combined SSM after 12 modes. The convergence of Scapular SSM Generalisation across the number of principal components has previously been reported to occur within 5 modes [9]; there is no published Generalisation ability data for proximal humerus or combined two-body models for comparison purposes.

### Model specificity

The comparison of model Specificities is similar to that of Generalisation abilities; the Proximal-Humerus SSM, by virtue of the simpler modeled shape, exhibits lesser training point distances than the more complex Scapula SSM, and the Combined SSM Specificity lies in between. Scapula SSM Specificity with 7 principal components, measured as mean RMS point distance to closest training set member, has previously been reported to be 3.79 mm [9]; in comparison, we report a mean *average Euclidean* training set point distance of 2.46 mm. There is no published Specificity data for proximal humerus or combined two-body models for comparison purposes.

### Model compactness

At least 90% of cumulative shape variation of the Proximal-Humerus and Scapula SSMs are described by the first 6 and 10 modes of variation respectively. The Proximal-Humerus SSM is less compact than those reported in the literature (requiring 4 [13], and 5 [12] modes to capture 90% cumulative shape variation), most likely owing to the pathologic shape variation that is additionally described by the Proximal-Humerus SSM whereas the others describe healthy populations. Note that typical full humerus SSMs [14, 15, 29] exhibit high compactness by virtue of the shape variance being dominated by humeral length, which is expressed in the first mode of variation, but the proximal humerus does not exhibit the same degree of length variation. The Scapula SSM compactness is within the range of that described in the literature with the number of components required to capture at least 90% cumulative shape variation reported between 5 and 11 modes [9, 14, 15, 29]. There is no published compactness data available for direct comparison with the Combined SSM. However, it is reasonable for the Combined SSM to exhibit lower compactness than both the Scapula and Proximal-Humerus SSMs, requiring a number of principal components to describe at least 90% of the cumulative shape variation that falls between that of the Scapula SSM (10) and the sum of those required by the Proximal-Humerus and Scapula SSMs (6 + 10 = 16). This is due to the relative shape complexity of the combination of both anatomical structures together, and the expectation of some degree of coupled variation between the two shapes.

### SSM modes of variation

The primary modes of variation have direct anatomical measurement interpretations, indicating that the models effectively capture meaningful features and that patients undergoing RTSA have measurable characteristic scapulohumeral bony anatomy shape variation.

The primary modes of variation observed in the Scapula SSM are; *Mode 1*: Overall Scaling, *Mode 2*: Aspect Ratio (medial–lateral width with commensurately decreased superior-inferior height) and Acromial Slope [3], *Mode 3:* Posterior Glenoid Erosion negatively correlated with Lateral Acromial Tip Projection, *Mode 4:* Acromial Tilt [3] and Posterior Coracoid Tip Shift, *Mode 5:* Inferior Glenoid Erosion negatively correlated with Posterior Acromial Shift, and *Mode 6*: Posterior Glenoid Erosion with Superior Spine Shift. The primary modes of variation observed in the Scapula SSM, compared to an excerpt from the literature, are presented in Table 9.

**Table 9:** Comparison of scapula ssm primary modes of variation

**Source**	**This Study**	**Sharif-Ahmadian et al. **[9]	**Soltanmohammadi et al.** [14]	**Casier et al.** [10]
Population	RTSA Patients	Walch B2 and B3	Healthy	Healthy
Mode 1	Overall Scaling	Homogeneous Size	Overall Scaling	Size
Mode 2	Aspect Ratio	Scapular Breadth	Rotation of Medial and Inferior Borders	Coraco-acromial Rotation
Mode 3	Glenoid Erosion, Acromial Tip	Glenoid Erosion	Superior-Inferior Length, Coracoacromial Elongation	Acromial Shape
Mode 4	Acromial Tilt, Coracoid Tip Shift	Acromial Tip Shift, Coracoid Shift	Infero-lateral Elongation, Coraco-acromial Tilt	Spine Shape
Mode 5	Glenoid Erosion, Acromial Shift	Acromial Shift	Coracoid Length, Acromial Tilt	Acromial Overhang
Mode 6	Glenoid Erosion, Spine Shift	Acromial Shift	Medial Border Tilt, Coracoid Tilt	

The primary modes of variation observed in the Proximal-Humerus SSM are: *Mode 1*: Overall Scaling, *Mode 2*: Lesser Tuberosity Prominence, *Mode 3*: Inferior Head Erosion negatively correlated with Greater Tuberosity Prominence, *Mode 4:* Medial Offset, *Mode 5*: Lateral Bowing, and *Mode 6*: Posterior Offset. The primary modes of variation observed in the Proximal-Humeral SSM, compared to an excerpt from the literature, are presented in Table 10.

**Table 10:** Comparison of proximal-humerus ssm primary modes of variation

**Source**	**This Study**	**Sintini et al.** [12]	**Casier et al. **[10]	**Sade **[13]
Population	RTSA Patients	Healthy	Healthy	Healthy
Humerus Portion	Proximal	Proximal	Full	Proximal
Mode 1	Overall Scaling	Uniform Scaling	Overall Scaling	Scaling
Mode 2	Lesser Tuberosity	Head Shape, Inclination, Medial Offset	Axial Elongation, Head Medial Rotation	Lesser Tuberosity
Mode 3	Head Erosion, Greater Tuberosity	Greater Tuberosity, Surgical Neck	Girth	Greater Tuberosity, Medial Neck Curvature
Mode 4	Medial Offset	Greater Tuberosity, Surgical Neck	Retroversion	
Mode 5	Lateral Bowing	Head Shape, Neck Orientation	Lateral Bowing of Shaft	
Mode 6	Posterior Offset	Posterior Offset, Greater Tuberosity, Surgical Neck		

The primary modes of variation observed in the Combined SSM are: *Mode 1*: Overall Scale of both the Scapula and Proximal Humerus and Humeral Head Inclination, *Mode 2*: Scapular Fulcrum Axis, Acromial Anterior Projection and Humeral Shaft Diameter and Head Radius of Rotation, *Mode 3*: Scapular Aspect Ratio, Posterior Glenoid Erosion and Version, Acromial Tilt, Inferior Angle Prominence, with Humeral Head Inclination, Erosion, Medial Offset, and Greater Tuberosity Angle.

### Quantification of coupled scapulohumeral variation

The scapular contributions to Combined SSM eigenvectors, ${{\varvec{A}}}_{S}$, are more ordered and less dispersed than those of the proximal humerus, ${{\varvec{A}}}_{H}$. This is because the training set scapulae exhibit greater absolute variation than the proximal humeri. As a result, the scapular variation predominates and dictates the ordering of the Combined SSM eigenvectors, with the proximal humerus variation incorporated into each Combined SSM eigenvector based on its coupling with the scapular variation. However, the Combined SSM eigenvectors do not match the Scapula SSM eigenvectors in lockstep (and so ${{\varvec{A}}}_{S}$ does not form a perfect diagonal matrix) as each Scapula SSM eigenvector can be coupled to multiple Proximal-Humerus SSM eigenvectors independently; a separate Combined SSM eigenvector is required to describe each independent Scapula-Proximal-Humerus eigenvector coupling. For example, the second Combined SSM eigenvector (represented by the second row of both heatmaps Fig. 16) comprises the second Scapular SSM mode of variation coupled with the *first* mode of variation of the Proximal-Humeral SSM. The third Combined SSM eigenvector (i.e. the third row of both heatmaps Fig. 16) also includes the second mode of variation from the Scapular SSM but is coupled with the *second* mode of variation of the Proximal-Humeral SSM.

This *smearing* of scapular variation that is captured in one principal component of the Scapula SSM across multiple principal components of the Combined SSM is what makes the Combined SSM comparatively less compact. The coupled shape variation between the scapula and proximal humerus is significant, with up to 6.21% of total study population variation coupled in a single Combined SSM eigenvector (Mode 3) and 43.2% of total variation coupled across all Combined SSM modes of variation. This significant coupling underscores the necessity for a combined two-body SSM in generative applications, as it reveals the violation of the assumption of shape independence that is inherent in the use of separate one-body SSMs.

### Prediction of missing counterpart

The covariance matrix between scapular and proximal-humeral contributions to Combined SSM model parameters is clearly sparse, but does indicate linear correlation between the earlier scapular contributions across most proximal-humeral contributions, and vice versa.

The relationship between scapular and proximal-humeral model parameters in the Combined SSM is successfully exploited in the missing body predictions with scapular average point distance error distribution median of 3.83 mm (IQR: 2.07 mm) compared to the Training Set Mean’s median of 4.26 mm (IQR: 1.64 mm) — a reduction in median error by more than 10%. Recall that Ground Truth Accuracy (Fig. 6) is a measure of an SSM’s accuracy in describing a shape that it is presented with, serving as a measure of the best possible prediction achievable (median Scapula SSM average point error of 1.08 mm). Under this interpretation, the Combined SSM improves upon the naive Training Set Mean prediction by 13.8% of the total possible prediction error reduction. This is a surprising display of bootstrapping whereby the relatively simple proximal humerus meaningfully enhances the prediction of the significantly more complex scapula.

The proximal humeri, by virtue of their relative simplicity and lesser variation, are generally more similar to the mean shape across the training set than were the scapulae. For this reason, the Combined SSM does not meaningfully alter median average prediction error from that of the Training Set Mean. However, the Combined SSM does reduce the prediction error interquartile range by over 9% with median average point distance error of 2.24 mm (IQR: 1.13 mm) compared to 2.21 mm (IQR: 1.24 mm) for the Training Set Mean.

To provide clinical context for the missing body prediction errors, arthroplasty glenoid implant pegs can have a diameter of approximately 3 mm [54], and glenoid implants are usually placed within 3 mm of the intended location when using patient-specific targeting guides [54, 55]. Implants are considered malpositioned if placed more than 4 mm from the intended location [56]. Therefore, use of the Combined SSM for missing body prediction reduces median error to a level comparable with acceptable glenoid implant placement accuracy.

### De Novo generation of synthetic populations

Noting that the *p*-value, or plausibility, distribution should be uniform for an unbiased shape synthesis process, we see that the distribution formed by the Combined SSM-generated shapes is approximately uniform with near perfect quartiles. Oppositely, the *p*-value distribution formed by shapes generated by combining the one-body SSMs is biased with excessive high *p*-value (highly plausible) shapes, resulting in median *p*-value of 0.55 and 75-th percentile of .82, but also demonstrating a large proportion of implausible shapes (i.e. low *p*-values).

The intuitive physical interpretation of the skewed distribution is that generating scapula-proximal-humerus shape pairs using two independent SSMs produces too many synthetic shapes that are too similar to the mean shape of the training set. In comparison, the Combined SSM generates a set of synthetic shapes that is more evenly distributed across the entire range of possibilities. More diverse synthetic shapes are more likely under the Combined SSM because its individual eigenvectors encode (coupled) shape variation of both bodies such that a single extreme model parameter ${b}_{C,i}$ begets significant variation in both the scapula and the proximal-humerus shapes. Randomly drawing a single, extreme, model parameter for the Combined SSM is more probable than drawing two separate extreme parameters—one for each of the independent SSMs—as would be necessary to generate a comparable synthetic shape pair under the independent SSM framework. This result is striking because intuitively we would expect two independent one-body models to produce mismatched, and thus low-plausibility, scapula-proximal-humerus pairs; while there is clearly a cluster of low-plausibility shapes that create a relative low-plausibility peak in the distribution for the two SSM-synthesized shapes, it is the aforementioned high-plausibility effect that dominates.

The Kolmogorov–Smirnov Test results quantifiably support the conclusion that the Combined SSM shape plausibilities are correctly distributed, and that those from the two independent one-body models are not. This means that the use of two independent one-body SSMs in generative applications, such as for computational studies of virtual surgeries [6, 57, 58], would introduce population bias that violates the underpinning assumptions of the work — their outputs are either too similar to the training set mean, or otherwise too implausible due to counterpart mismatch (ignored coupled variation), and therefore do not accurately capture the expected variation of shapes across a real clinical population.

### Prospective influence on surgical decision-making and planning

The combined two-body SSM technology exemplified in this study facilitates enhanced evidence-based RTSA design parameter selection and prosthesis placement. Similarly, it informs the design of patient-specific instrumentation to improve component positioning and fixation [59]. Traditional biomechanical analysis of the shoulder has been limited by either small sample sizes—due to the labor-intensive nature of manual segmentation—or the need to assume a population-averaged humeral shape when analyzing scapular anatomy. These constraints introduce bias and reduce the fidelity of joint-level analysis, particularly in patients with atypical or pathologic anatomy. In contrast, the combined two-body SSM developed in this study enables comprehensive and anatomically grounded biomechanical evaluation through the synthesis of coupled scapular and humeral geometries. This model supports next-generation computational analyses capable of informing optimal glenosphere sizing, humeral offset and inclination angle, component lateralization, and implant positioning strategies for RTSA. Such simulations can be scaled to large synthetic cohorts, enabling robust design evaluations that reflect real-world anatomic variation [6, 9].

The developed SSM also offers a novel method to quantify disease severity, offering an objective basis for triage and supporting the identification of surgical indications in clinical decision-making. Mode 3 of the combined two-body SSM encodes the coupled pattern of glenoid erosion and humeral head erosion commonly observed in arthropathic shoulders. Leveraging this, a future study could investigate the appropriateness of using the z-score (the number of standard deviations from the mean) of a shape pair along Mode 3 of the SSM as a novel quantification of disease severity that compactly describes multiple simultaneous dimensions of pathologic shape variation. This score serves as a novel unified metric that simultaneously describes glenoid retroversion, glenoid erosion, and humeral head erosion; in the absence of this composite score, multiple individual measurements are needed, complicating both interpretation and the ability to systematically rank subjects along this multidimensional variation. This score could be integrated into artificial intelligence-powered clinical decision support systems to assist in triaging patients, informing thresholds for surgical intervention, or tracking radiographic disease progression. Moreover, the model’s ability to quantify anatomy along a continuum of degeneration may serve as a foundation for predictive algorithms assessing future joint deterioration or likelihood of requiring arthroplasty.

Another area of significant potential application for this combined two-body SSM is in guiding complex reconstruction in the setting of comminuted fractures. In cases of severe trauma, with comminuted proximal humeral or scapular fractures, the reconstruction of the original anatomy is often impeded by incomplete bony structures. The combined two-body SSM provides a powerful tool for generating plausible patient-specific pre-injury geometry by statistically inferring missing anatomy based on intact bone structures. This functionality is particularly useful for designing patient-specific fixation strategies or implants in cases where inference of exact original anatomy is not feasible. The model's coupled variation ensures anatomical coherence between reconstructed segments, thereby improving the accuracy and functional outcomes of complex shoulder reconstructions. Similar AI-assisted preoperative planning in the context of total hip arthroplasty has already demonstrated superior lower-limb length restoration compared to traditional methods [60].

In addition to its utility in planning and reconstruction, the combined two-body SSM may also support automated radiographic identification of glenohumeral arthropathy. By capturing the hallmark features of posterior glenoid wear and associated humeral head erosion within its coupled modes of variation, the model offers a data-driven basis for classifying patients according to established arthritic subtypes. Integrated into a radiographic workflow, this could enable the automated flagging of arthropathic morphology on standard shoulder imaging, reducing diagnostic variability and expediting the clinical decision-making process. Such automation aligns with recent advances in musculoskeletal imaging, where deep learning tools have demonstrated performance comparable to orthopaedic surgeons and radiologists [61] in detecting fractures [62–64], dislocations [64], osteoarthritis [64], and rotator cuff degeneration [65, 66].

### Limitations

This study is limited by the small sample size of only 45 patients. However, the analysis of the model’s Generalization ability across different training set sizes revealed no significant improvement in performance beyond 18 training samples for any SSM. The use of manual segmentation and measurement of underlying CT imaging data by a single investigator, though reviewed by a second investigator, introduces potential for human error and variability.

## Conclusions

This study presents the first statistical shape models of the proximal humerus and the combined two-body glenohumeral joint derived from a clinically pathologic population. The interrelationship between scapula and proximal humerus geometry is effectively captured by the Combined SSM, enabling enhanced missing body predictions in scenarios where complete imaging is unavailable. Notably, Mode 3 of the Combined model uniquely characterizes pathologic glenohumeral variation, offering potential applications in early automated disease detection, disease progression prediction, pathogenetic analysis, and the generation of pathologic synthetic populations. The necessity of combined two-body models for generating unbiased synthetic glenohumeral populations is clearly demonstrated, positioning this model as a valuable tool for advancing computational studies in shoulder biomechanics and surgical planning.

## Data Availability

The datasets generated during the current study are available from the corresponding author on reasonable request. The datasets analyzed during the current study are not publicly available due institutional privacy requirements but are available from the corresponding author on reasonable request.

## Abbreviations

CT: Computed Tomography
IQR: Interquartile Range
PCA: Principal Component Analysis
RTSA: Reverse Total Shoulder Arthroplasty
SSM: Statistical Shape Model
